# Hemodynamic factors primarily impact on carotid IMT in young adults of African Ancestry in Sub-Saharan Africa

**DOI:** 10.1038/s41371-026-01119-8

**Published:** 2026-02-07

**Authors:** Nico Malan, Gavin R. Norton, Vernice R. Peterson, Nonhlanhla H. Mthembu, Carlos D. Libhaber, Andrea Kolkenbeck-Ruh, Grace Tade, Pinhas Sareli, Patrick H. Dessein, Angela J. Woodiwiss

**Affiliations:** From the Cardiovascular Pathophysiology and Genomics Research Unit, Department of Physiology, School of Biomedical Sciences, Faculty of Health Sciences, University of the Witwatersrand, Johannesburg, South Africa

**Keywords:** Risk factors, Medical imaging

## Abstract

Cardiovascular events occur 20 years earlier in Sub-Saharan Africa compared to Europe. The risk factors for atherosclerosis differ between population groups and according to age. We compared the main correlates of carotid intima-media thickness (IMT, an index of atherosclerosis) in young and older adults of African ancestry. Hemodynamic (central and peripheral arterial pressures) and metabolic factors (lipids, glucose, glycated haemoglobin), smoking status and carotid IMT were determined in 573 adult Africans. In young (age<35years, n = 181) and middle-aged (35–59years, n = 231) adults, carotid IMT was associated with hemodynamic and metabolic cardiovascular risk factors on bivariate analyses. In older (age≥60years, n = 161) adults only hemodynamic factors were associated with carotid IMT. After adjustments for confounders, lipids were not associated with carotid IMT at any adult age. Carotid IMT was independently associated with backward wave pressure (Pb, p = 0.001) and age (p = 0.006) in young adults; with hemodynamics (central systolic blood pressure, p = 0.003; Pb, p = 0.02), age (p = 0.0002), body mass index (BMI, p = 0.005) and heart rate (p = 0.007) in middle-aged adults; and with Pb (p < 0.0001), male sex (p = 0.03), and HR (p = 0.04) in older adults. Increased carotid IMT was related to Pb in young (odds ratio [OR] = 1.233, p = 0.0003) and older (OR = 1.086, p = 0.0059) adults, and BMI (OR = 1.089, p = 0.0005) in middle-aged adults. Improvements in predictive performance for detecting increased carotid IMT were shown with Pb in young (p = 0.0032) and older (p = 0.0031) adults, and with BMI (p = 0.0004) in middle-aged adults. In conclusion, in African adults in Sub-Saharan Africa, carotid IMT is associated with hemodynamic factors, but not lipids. Moreover, in young adults, carotid IMT is primarily associated with hemodynamic factors.

## Introduction

Cardiovascular disease (CVD) is the primary cause of death worldwide [1]. However, there are differences in the burden of CVD between countries, with concerns of an emerging pandemic of CVD in Sub-Saharan Africa [2], in contrast to Europe and America, where CVD appears to be on the decline [3]. Moreover, in people of African descent, the onset of cardiovascular outcomes is premature, occurring approximately 15–20 years earlier than in people of European descent [4]. Hence, it is essential to screen for and prevent CVD in Sub-Saharan Africa, especially among young adults.

Carotid artery intima-media thickness (IMT), a proxy for subclinical atherosclerosis, is an important non-invasive screening tool for CVD [5]. However, carotid IMT is lower in African populations living in Africa compared to other populations [6]. Variations in risk factors for CVD in Sub-Saharan African compared to European and American countries, may account for population differences in IMT. A recent systematic review points toward hypertension rather than dyslipidaemia, being the primary risk factor for CVD in Sub-Saharan African countries [7]. Indeed, central arterial stiffness, central arterial blood pressure (BP) [8] and central arterial pressure augmentation [9] are elevated in African compared with European individuals, particularly among young adults [8]. Moreover, in African populations living in Africa compared to in European populations, HDL cholesterol has a greater protective effect and LDL cholesterol a weaker negative impact on carotid IMT [6].

Despite the high prevalence of CVD, there are limited data assessing the determinants of carotid IMT in Sub-Saharan African populations, especially in young adults. Although a large study in four Sub-Saharan African countries identified factors associated with carotid IMT, these data were restricted to adults aged 40–60 years [10]. In this regard, the determinants of carotid IMT have been shown to differ according to age in Chinese [11], and European [12] individuals. However, to our knowledge there are no data assessing the determinants of carotid IMT in young adults of African ancestry. We therefore aimed to identify the main hemodynamic and metabolic cardiovascular risk factors associated with carotid IMT in young (age<35 years) compared to middle-aged (age 35–59 years) and older (age≥60 years) adults of African ancestry.

## Methods

### Study group

The present study was approved by the Committee for Research on Human Subjects of the University of the Witwatersrand (approval number: M02-04-72 and renewed as M07-04-69, M12-04-108, M17-04-01, and M22-03-93), and conducted according to the principles outlined in the Helsinki declaration. Participants gave informed, written consent. The design of the study has previously been described [13, 14]. Briefly, families of black African descent (Nguni and Sotho chiefdoms) with siblings older than 16 years of age were randomly recruited from the South West Township (SOWETO) of Johannesburg, South Africa. In the present study, 573 participants with both carotid IMT and high quality velocity measurements in the aortic outflow tract were assessed. To identify the cardiovascular risk factors associated with carotid IMT in young compared to older adults, the study group was divided into three age categories: young: age<35 years (n = 181); middle-aged: age 35–59 years (n = 231); elderly: age≥60 years (n = 161).

### Clinical, demographic, anthropometric and laboratory information

Demographic, lifestyle and clinical data were obtained by means of a questionnaire [13]. Standard approaches were used to measure height and weight. Participants were considered to be overweight if their body mass index (BMI) was ≥25 kg/m^2^ and obese if their BMI was ≥30 kg/m^2^. Blood glucose, lipid profiles, and percentage glycated hemoglobin (HbA1c) were assessed after at least a 12 h fast. Diabetes mellitus (DM) was defined as an HbA1c value greater than 6.5%, or the use of insulin or oral glucose lowering agents. We also determined fasting plasma insulin concentrations from an insulin immulite, solid phase, two-site chemiluminescent immunometric assay (Diagnostic Products Corporation, Los Angeles, CA, USA) and insulin resistance was estimated by the homeostasis model assessment of insulin resistance (HOMA-IR) using the formula (insulin [µU/ml] × glucose [mmol/l])/22.5. High quality office brachial BP measurements were obtained according to guidelines, after 5 min of rest in the seated position, by a trained nurse-technician using a standard mercury sphygmomanometer [13]. Office BP was the mean of 5 brachial BP measurements obtained at least 30 s apart. Hypertension was defined as a mean office systolic BP ≥ 140 mm Hg or diastolic BP ≥ 90 mm Hg or the use of antihypertensive medication. Uncontrolled BP was defined as mean office systolic BP ≥ 140 mm Hg or diastolic BP ≥ 90 mm Hg.

### Carotid IMT

Carotid IMT was determined using high resolution B-mode ultrasound (SonoCalc IMT, Sonosite Inc, Bothell, Washington) as previously described [15] employing a linear array 7.5 MHz probe. Images of at least 1 cm length of the far wall of the distal portion of both the right and left common carotid arteries from an optimal angle of incidence (defined as the longitudinal angle of approach where both branches of the internal and external carotid artery are visualised simultaneously) at least 1 cm proximal to the flow divider were obtained. Carotid IMT measurements were determined using semi-automated border-detection and quality control software [15]. At least 3 measurements were obtained from both the right and left sides and the mean of data from both sides was used for analyses. Increased IMT was defined as >75^th^ percentile for decade of age and sex as previously defined in normotensive, nondiabetic participants [15]. Carotid plaque was determined from both longitudinal and cross-sectional images in and around the carotid bifurcation [15]. Carotid plaque was defined according to the Mannheim consensus as a focal structure that encroaches into the arterial lumen of at least 0.5 mm or 50% of the surrounding IMT value or demonstrates a thickness >1.5 mm as measured from the media-adventitia interface to the intima-lumen interface.

### Hemodynamic assessments

Hemodynamic parameters were determined from non-invasive central arterial pressure measurements derived from peripheral pulse wave analysis (radial artery applanation tonometry and SphygmoCor software), and the assessment of aortic velocity and diameter in the outflow tract (Acuson SC2000 Diagnostic ultrasound system, Siemens Medical Solutions, USA, Inc.) as previously described [14, 16].

After participants had rested for 15 min in the supine position, arterial waveforms at the radial (dominant arm) pulse were recorded, during an 8 s period, by applanation tonometry using a high-fidelity SPC-301 micromanometer (Millar Instrument, Inc., Houston, Texas). The micromanometer was interfaced with a computer employing SphygmoCor, version 9.0 software (AtCor by Cardiex, Sydney, New South Wales, Australia). The pulse wave was calibrated by manual measurement (auscultation) of brachial systolic and diastolic BP taken immediately before the recordings. A validated generalised transfer function incorporated in SphygmoCor software was used to convert the peripheral pressure waveform into a central aortic waveform. Recordings where the systolic or diastolic variability of consecutive waveforms exceeded 5% or the amplitude of the pulse wave signal was less than 80 mV, were discarded.

Immediately after obtaining the central arterial pressure waveforms, aortic velocity and diameter measurements were acquired by an experienced observer (AJW) using an Acuson SC2000 Diagnostic ultrasound system (Siemens Medical Solutions, USA, Inc.). High quality velocity assessments, obtained in the 5-chamber view, were identified as those with a smooth velocity waveform, a dense leading (outer) edge and a clear maximum velocity. Aortic diameter measurements were obtained just proximal to the aortic leaflets in the long axis parasternal view, and the largest diameter recorded in early systole was used to construct the aortic flow waveform. Taking care to avoid any overshoot of the image, the leading (outer) edge or the most dense, or brightest portion of the spectral image of the velocity waveform was outlined using graphics software. The velocity waveform and aortic diameter measurements were employed to construct an aortic flow waveform where flow = velocity x aortic root cross-sectional area calculated from diameter assessments. Stroke volume (SV) was calculated from the product of the velocity-time integral and aortic root cross-sectional area. Stroke volume was also indexed to body surface area. MAP was determined from the arterial pressure wave using SphygmoCor software. Total arterial compliance (TAC) was calculated as SV/PPc. As it has been recommended that either time or frequency domains are appropriate for the assessment of aortic characteristic impedance (Zc) [17], Zc was determined in the time domain using approaches previously described [14, 16, 18] and validated against invasive pressure measurements [19]. The volume flow waveform was paired with the central arterial pressure waveform by aligning the foot (t_0_) of the respective signal averaged waveforms. The point at which flow achieves 95% of its peak (t_Q95_) was identified. The change in pressure between t_0_ and t_Q95_ was determined. Aortic characteristic impedance was calculated as the ratio of change in pressure to change in flow in the window t_0_ to t_95_. Wave separation analysis was performed using Zc values and flow and pressure waveforms, and backward wave pressures (Pb) determined from (PPc - peak P_QxZc_)/2. The contribution to forward wave pressures (Pf) of pressures determined by an interaction between flow (Q) and Zc was identified from the pressures generated by the product of peak aortic Q and Zc (peak P_QxZc_) [20]. The use of peak P_QxZc_ rather than Pf, excludes the possibility of errors inherent in the use of Pf which includes pressures generated by wave re-reflection [20].

### Data analysis

Database management and statistical analyses were performed with SAS software, version 9.4 (The SAS Institute, Cary, NC). Continuous variables are expressed as mean ( ± SD) for parametric data or median (interquartile range) for non-parametric data. Dichotomous variables are expressed as percentages. As glycated haemoglobin, blood glucose, HOMA-IR, total, LDL, HDL and total/HDL cholesterol were not normally distributed they were logarithmically transformed (natural logarithm, ln) prior to performing linear regression analyses. To identify independent relationships between carotid IMT and hemodynamic and metabolic factors, multivariate adjusted linear regression analyses were performed. Adjustments were for age, sex, BMI, DM (except for glucose due to collinearity), regular alcohol intake, regular tobacco intake, heart rate, treatment for hypertension and MAP (except for hemodynamic factors due to collinearity). Logistic regression analysis was performed to determine the odds of increased carotid IMT in association with one unit increments in hemodynamic or metabolic factors independent of confounders. The performance of hemodynamic or metabolic factors in identifying individuals with increased carotid IMT was assessed using receiver operator characteristic (ROC) curve analysis, and the determination of integrated discrimination improvement, and net reclassification improvement [21, 22].

## Results

### Participant characteristics

Table S1 (on-line supplement) shows the general characteristics of all participants in the current study. A large proportion were either overweight or obese, almost a half had hypertension, which was largely uncontrolled, over a third had increased carotid IMT, but the prevalence of plaque was low (< 5%). Almost a half of the women were postmenopausal, however, none of the women were receiving hormone replacement therapy.

Table 1 shows the general characteristics of the participants according to age group. As per definition, the 3 groups differed by age. The young adults had less obesity, hypertension and DM, lower blood glucose, glycated hemoglobin, total and LDL cholesterol, triglycerides, BP, SV, Pb, Q, peak P_QxZc_, carotid IMT and plaque than the middle-aged and older groups. In addition, the middle-age group had less hypertension and DM, lower blood glucose, glycated hemoglobin, total and LDL cholesterol, triglycerides, BP, SV, Pb, Q, peak P_QxZc,_ carotid IMT, and plaque than the older age group.

**Table 1:** Characteristics of study participants according to age group.

	Young ( < 35years)	Middle-aged (35-59years)	Older ( ≥ 60years)
Sample size	181	231	161
% women (n)	61.9 (112)	73.6 (170)*	71.4 (115)*
% postmenopausal women (n)	0 (0)	45.3 (77)***	100.0 (115)*** ^†††^
Age (years)	25.9 ± 5.1	48.3 ± 6.8***	68.9 ± 7.0***^†††^
Body mass index (kg/m^2^)	25.6 ± 6.0	32.0 ± 7.6***	32.2 ± 7.2***
% Overweight (n)	27.6 (50)	22.5 (52)	25.5 (41)
% Obese (n)	20.4 (37)	58.9 (136)***	57.8 (93)***
% Hypertensive (n)	16.6 (30)	48.1 (111)***	77.0 (124)*** ^†††^
% Treated for hypertension (n)	2.2 (4)	26.8 (62)***	57.1 (92)*** ^†††^
% Hypertensives treated for hypertension (n)	13.3 (4/30)	55.9 (62/111)***	74.2 (92/124)*** ^†††^
% Uncontrolled BP (n)	14.9 (27)	35.9 (83)***	53.4 (86)*** ^†††^
% Hypertensives with uncontrolled BP (n)	90.0 (27/30)	74.8 (83/111)	69.4 (86/124)*
% Regular smoking (n)	22.1 (40)	11.7 (27)**	11.8 (19)**
% Regular alcohol (n)	24.9 (45)	20.8 (48)	14.9 (24)
% Diabetes mellitus (n)	0.6 (1)	11.7 (27)***	26.1 (42)*** ^†††^
Carotid intima media thickness (IMT) (mm)	0.54 ± 0.06	0.65 ± 0.12***	0.76 ± 0.13***^†††^
% Increased IMT (n)	32.0 (58)	35.1 (81)	47.2 (76)
% Plaque (n)	0.55 (1)	3.46 (8)*	10.56 (17)***^††^
Metabolic Factors (Fasting plasma concentrations)			
Glucose (mmol/l)	4.40 (4.00 to 4.70)	4.60 (4.30 to 5.10)***	5.00 (4.60 to 5.80)*** ^†††^
Glycated haemoglobin (%)	5.60 (5.50 to 5.80)	5.88 (5.50 to 6.10)**	6.09 (5.79 to 6.54)*** ^††^
Insulin (μU/ml)	6.53 (4.54 to 12.00)	6.89 (4.59 to 12.74)	7.42 (3.88 to 12.40)
HOMA-IR	1.27 (0.82 to 2.35)	1.57 (0.91 to 2.86)*	1.73 (0.84 to 3.11)*
Total cholesterol (mmol/l)	4.09 (4.00 to 4.49)	4.63 (4.49 to 5.16)***	5.23 (4.70 to 5.40)*** ^†††^
LDL cholesterol (mmol/l)	2.24 (2.15 to 2.57)	2.70 (2.50 to 3.07)***	3.19 (2.80 to 3.26)*** ^†††^
HDL cholesterol (mmol/l)	1.41 (1.30 to 1.50)	1.40 (1.20 to 1.46)	1.36 (1.24 to 1.40)*
Triglycerides (mmol/l)	0.80 (0.70 to 1.00)	1.10 (0.95 to 1.47)***	1.52 (1.30 to 1.62)*** ^†††^
Total/HDL cholesterol ratio	2.88 (2.73 to 3.25)	3.50 (3.12 to 3.73)***	3.85 (3.75 to 4.08)*** ^†††^
Hemodynamic factors			
Brachial systolic BP (SBP) (mm Hg)	116 ± 15	127 ± 19***	139 ± 22***^†††^
Brachial diastolic BP (mm Hg)	77 ± 13	84 ± 12***	85 ± 11***
Brachial pulse pressure (mm Hg)	38.2 ± 9.1	42.1 ± 12.1**	53.8 ± 16.4***^†††^
Mean arterial pressure (MAP) (mm Hg)	90 ± 13	100 ± 14***	104 ± 14***^††^
Central arterial systolic BP (SBPc) (mm Hg)	105 ± 15	120 ± 19***	130 ± 21***^†††^
Central arterial pulse pressure (PPc) (mm Hg)	26.9 ± 7.4	33.9 ± 10.7***	44.6 ± 14.9***^†††^
Heart rate (beats/min)	66.4 ± 11.9	67.6 ± 11.4	68.3 ± 13.8
Peak aortic flow (Q) (mls/sec)	297 ± 126	340 ± 169*	405 ± 256***^††^
Stroke volume (SV) (mls/beat)	67.5 ± 30.7	80.9 ± 39.5**	91.2 ± 39.4***^††^
Stroke volume (mls/beat.BSA)	39.6 ± 17.4	43.6 ± 21.2*	50.6 ± 22.6***^††^
Aortic characteristic impedance (Zc)(dynes.cm^-5^)	85.5 ± 40.9	81.8 ± 45.1	92.9 ± 52.1^†^
Total arterial compliance (TAC) (mm Hg/mls.bt)	2.64 ± 1.29	2.55 ± 1.37	2.26 ± 1.22*^†^
Backward wave pressure (Pb) (mmHg)	9.8 ± 3.3	12.9 ± 5.1***	16.5 ± 6.4***^†††^
Peak P_QxZc_ (mm Hg)	22.2 ± 5.4	23.9 ± 7.2*	29.9 ± 9.3***^†††^

Data are shown as mean ± SD, proportions, or median and interquartile range. BP, blood pressure; BSA, body surface area; HDL, high-density lipoprotein; HOMA-IR, homeostatic model assessment for insulin resistance; LDL, low-density lipoprotein; Peak P_QxZc_, component of forward wave pressure generated by the product of peak Q and Zc.

*p < 0.05.

**p < 0.005.

***p < 0.0001 versus young; ^†^p < 0.05, ^††^p < 0.005; ^†††^p < 0.0001 versus middle-aged.

### Unadjusted associations between cardiovascular risk factors and carotid IMT in all participants

On-line supplemental Table S2 shows the bivariate associations between carotid IMT and cardiovascular risk factors, including hemodynamic and metabolic factors, in all participants. Sex, regular smoking, and regular drinking were the only factors not associated with carotid IMT. Although, on bivariate analysis in women only, postmenopausal status was associated with carotid IMT (Pearson’s r = 0.592, p < 0.0001), after adjustment for age this relationship was no longer significant (Partial r = 0.066, p = 0.19). Furthermore, after inclusion of multiple cardiovascular risk factors (age, BMI, smoking, alcohol, diabetes mellitus, treatment for hypertension, mean arterial pressure, and heart rate), postmenopausal status was not associated with carotid IMT (p = 0.17).

### Unadjusted associations between cardiovascular risk factors and carotid IMT in different age groups

Table 2 shows the bivariate associations between carotid IMT and cardiovascular risk factors, including hemodynamic and metabolic factors, in the three age groups. Regular smoking, regular drinking and treatment for hypertension were not associated with carotid IMT at any age. Of the metabolic risk factors, HOMA-IR, total cholesterol and LDL cholesterol were not related to carotid IMT in any of the age groups. Of the hemodynamic factors, blood flow (SV and Q) was not related carotid IMT in any age group.

**Table 2:** Bivariate associations between cardiovascular risk factors and carotid intima-media thickness (IMT) in three age groups.

IMT versus	Young ( < 35years) (n = 181)			Middle-aged (35-59years) (n = 231)			Older ( ≥ 60years) (n = 161)		
	r	(95% CI)	p-value	r	(95% CI)	p-value	r	(95% CI)	p-value
Age	**0.27**	**(0.13 – 0.40)**	**=0.0002**	**0.36**	**(0.24 – 0.47)**	**<0.0001**	**0.21**	**(0.05 – 0.35)**	**=0.0084**
Sex (male)	0.09	(−0.06 – 0.23)	=0.231	0.04	(−0.09 – 0.17)	=0.515	**0.17**	**(0.01 – 0.31)**	**=0.0356**
BMI	0.11	(−0.03 – 0.26)	=0.126	**0.22**	**(0.09 – 0.34)**	**=0.0007**	−0.08	(−0.23 – 0.08)	=0.321
Hypertension	**0.15**	**(0.01 – 0.29)**	**=0.0460**	**0.21**	**(0.08** – **0.33)**	**=0.0015**	0.10	(−0.05 – 0.25)	=0.188
Regular smoking	−0.09	(−0.23 – 0.06)	=0.232	0.04	(−0.09 – 0.17)	=0.577	−0.10	(−0.06 – 0.25)	=0.215
Regular alcohol	−0.01	(−0.16 – 0.14)	=0.884	−0.04	(−0.17 – 0.09)	=0.564	−0.04	(−0.11 – 0.20)	=0.580
Diabetes mellitus	−0.01	(−0.16 – 0.14)	=0.900	**0.14**	**(0.02 – 0.27)**	**=0.0276**	−0.07	(−0.22 – 0.09)	=0.401
Treatment for HT	0.12	(−0.03 – 0.26)	= 0.107	0.08	(−0.05 – 0.20)	=0.242	−0.01	(−0.16 – 0.14)	=0.896
Metabolic Factors									
Ln glucose	0.07	(−0.08 – 0.22)	=0.351	**0.21**	**(0.08 – 0.33)**	**=0.0021**	−0.06	(−0.21 – 0.09)	=0.439
Ln glycated haemoglobin	0.09	(−0.09 – 0.26)	=0.325	**0.16**	**(0.01 – 0.31)**	**=0.0474**	−0.13	(−0.32 – 0.07)	=0.207
Ln HOMA-IR	0.12	(−0.03 – 0.27)	=0.118	0.10	(−0.03 – 0.23)	=0.137	−0.05	(−0.21 – 0.11)	=0.507
Ln total cholesterol	0.06	(−0.09 – 0.21)	=0.408	0.04	(−0.09 – 0.17)	=0.534	−0.03	(−0.18 – 0.13)	=0.745
Ln LDL cholesterol	0.05	(−0.09 – 0.20)	=0.471	0.10	(−0.03 – 0.23)	=0.124	0.07	(−0.08 – 0.22)	=0.362
Ln HDL cholesterol	−0.11	(−0.26 – 0.03)	=0.125	−**0.15**	**(**−**0.27 – 0.02)**	**=0.0238**	−0.06	(−0.21 – 0.10)	=0.451
Ln Triglycerides	0.12	(−0.03 – 0.26)	=0.106	**0.18**	**(0.05 – 0.30)**	**=0.0071**	−0.09	(−0.24 – 0.07)	=0.267
Ln Total/HDL cholesterol	**0.16**	**(0.01 – 0.30)**	**=0.0320**	**0.18**	**(0.05 – 0.30)**	**=0.0067**	0.04	(−0.12 – 0.19)	=0.620
Hemodynamic factors									
Brachial systolic BP	0.13	(−0.02 – 0.27)	=0.088	**0.29**	**(0.17 – 0.40)**	**<0.0001**	**0.24**	**(0.09 – 0.38)**	**=0.0022**
Brachial diastolic BP	0.09	(−0.05 – 0.24)	=0.203	**0.20**	**(0.07 – 0.32)**	**=0.0025**	0.08	(−0.07 – 0.23)	=0.300
Brachial pulse pressure	0.09	(−0.05 – 0.24)	=0.206	**0.28**	**(0.16 – 0.40)**	**<0.0001**	**0.29**	**(0.14 – 0.43)**	**=0.0002**
Mean arterial pressure	0.12	(−0.02 – 0.26)	=0.101	**0.25**	**(0.13 – 0.37)**	**<0.0001**	**0.18**	**(0.02 – 0.32)**	**=0.0256**
Central arterial SBP	**0.15**	**(0.01 – 0.29)**	**=0.0436**	**0.28**	**(0.15 – 0.39)**	< **0.0001**	**0.23**	**(0.08 – 0.37)**	**=0.0033**
Central arterial PP	0.13	(−0.01 – 0.27)	=0.070	**0.26**	**(0.14 – 0.38)**	**<0.0001**	**0.29**	**(0.14 – 0.42)**	**=0.0002**
Heart rate	0.01	(−0.14 – 0.15)	=0.923	**0.14**	**(0.01 – 0.26)**	**=0.0388**	0.10	(−0.05 – 0.25)	=0.191
Peak aortic flow	0.01	(−0.14 – 0.15)	=0.910	0.02	(−0.10 – 0.15)	=0.707	0.02	(−0.14 – 0.17)	=0.831
Stroke volume	0.10	(-0.04 - 0.24)	=0.167	0.06	(−0.07 – 0.18)	=0.384	0.02	(−0.13 – 0.18)	=0.770
Stroke volume/BSA	0.06	(−0.08 – 0.21)	=0.393	0.01	(−0.11 – 0.14)	=0.835	0.03	(−0.13 – 0.18)	=0.707
Zc	−0.01	(−0.15–0.14)	=0.952	0.13	(−0.01 – 0.25)	=0.0554	**0.22**	**(0.07 – 0.36)**	**=0.0051**
TAC	0.01	(−0.14 – 0.15)	=0.903 **–0.14**	**(**−**0.26 – 0.01)**	**=0.0325**	−0.13	(−0.28 – 0.03)	=0.108	
Pb	**0.24**	**(0.10 – 0.38)**	**=0.0008**	**0.23**	**(0.10 – 0.35)**	= **0.0004**	**0.31**	**(0.17–0.44)**	**<0.0001**
Peak P_QxZc_	0.09	(−0.05 –0.23)	=0.218	**0.26**	**(0.14 –0.38)**	**<0.0001**	**0.30**	**(0.15 –0.43)**	**<0.0001**

Significant associations are shown in bold type.

*BMI* body mass index, *BP* blood pressure, *BSA* body surface area, *CI* confidence interval, *HDL* high-density lipoprotein, *HOMA-IR* homeostatic model assessment for insulin resistance, *HT* hypertension, *LDL* low-density lipoprotein, *Ln*, natural logarithm, *Pb* backward wave pressure, *PP* pulse pressure; Peak P_QxZc_, component of forward wave pressure generated by the product of peak Q and Zc; r=Pearson’s correlation coefficient; SBP, systolic blood pressure; TAC, total arterial compliance; Zc, aortic characteristic impedance.

In the young adults, prior to any adjustments, carotid IMT was associated with age and the presence of hypertension. Central systolic BP and Pb were the only hemodynamic factors, and total/HDL cholesterol was the only metabolic factor associated with carotid IMT in young adults (Table 2).

In middle-aged adults, prior to any adjustments, carotid IMT was associated with age, BMI, the presence of hypertension, and the presence of DM. The hemodynamic factors associated with carotid IMT were brachial systolic, diastolic and mean BP, brachial pulse pressure, central systolic BP, central pulse pressure, heart rate, TAC, Pb and peak P_QxZc_. The metabolic factors associated with carotid IMT in the middle-aged adults were blood glucose, glycated haemoglobin, total/HDL cholesterol, HDL cholesterol and triglycerides (Table 2).

In older aged adults, prior to any adjustments, carotid IMT was associated with age and male sex. Brachial systolic and mean BP, brachial pulse pressure, central systolic BP, central pulse pressure, Zc, Pb and peak P_QxZc_ were the hemodynamic factors associated with carotid IMT in older adults (Table 2). None of the metabolic factors were associated with carotid IMT in older adults (Table 2).

### Associations between carotid IMT and hemodynamic and metabolic factors independent of confounders in all study participants

On-line supplemental Table S3 shows the multivariate associations between carotid IMT and hemodynamic and metabolic cardiovascular risk factors independent of confounders, in all study participants. Prior to the inclusion of hemodynamic or metabolic factors in the model, age was the primary determinant of carotid IMT, with mean arterial pressure, male sex, heart rate and BMI also showing significant associations (Table S3). In addition, age remained associated with carotid IMT independent of both hemodynamic and metabolic factors (Table S3). In separate models, none of the metabolic factors were associated with carotid IMT independent of confounders including age (Table S3). However, a number of hemodynamic factors (brachial systolic BP, brachial pulse pressure, central arterial systolic BP, central arterial pulse pressure, Zc, Pb, peak P_QxZc_, and TAC) were significantly associated with carotid IMT independent of confounders including age (Table S3). Furthermore, when included in the same model central arterial PP or Pb, showed strong associations with carotid IMT independent of confounders including age, whereas total/HDL cholesterol, was only weakly associated with carotid IMT (Table S3).

### Associations between carotid IMT and hemodynamic and metabolic factors independent of confounders in different age groups

Table 3 (young), 4 (middle-aged) and 5 (older aged) show the multivariate associations between carotid IMT and hemodynamic and metabolic cardiovascular risk factors independent of confounders, in the three age groups. In young adults, prior to the inclusion of hemodynamic or metabolic factors in the model, age was the only determinant of carotid IMT (Table 3). In addition, age remained associated with carotid IMT independent of both hemodynamic and metabolic factors (Table 3). In separate models, total/HDL cholesterol and central arterial systolic BP were not associated with carotid IMT independent of confounders, whereas Pb remained associated with carotid IMT independent of confounders (Table 3). Furthermore, when included in the same model Pb, but not total/HDL cholesterol, was associated with carotid IMT (Table 3).

**Table 3:** Multivariate models showing associations between cardiovascular risk factors and carotid intima-media thickness (IMT) in young adults (age <35 years, n = 181).

IMT versus	Stand. β±sem	p-value	Stand. β±sem	p-value	Stand. β±sem	p-value	Stand. β±sem	p-value
	Model 1		Model 2		Model 3		Model 4	
Age	**0.238** ± **0.084**	=**0.0050**	**0.229** ± **0.084**	=**0.0069**	**0.234** ± **0.084**	=**0.0057**	**0.234** ± **0.081**	=**0.0042**
Sex (male)	0.111 ± 0.090	=0.219	0.102 ± 0.090	=0.261	0.103 ± 0.088	=0.243	0.145 ± 0.083	=0.082
BMI	0.025 ± 0.085	=0.767	0.008 ± 0.086	=0.926	0.018 ± 0.085	=0.835	0.002 ± 0.082	=0.983
Regular smoking	0.047 ± 0.083	=0.575	0.034 ± 0.084	=0.684	0.044 ± 0.083	=0.602	0.026 ± 0.081	=0.747
Regular alcohol	−0.055 ± 0.081	=0.496	−0.035 ± 0.083	=0.669	−0.053 ± 0.081	=0.517	−0.067 ± 0.079	=0.398
Diabetes mellitus	0.009 ± 0.074	=0.908	0.010 ± 0.074	=0.892	0.008 ± 0.074	=0.913	0.026 ± 0.072	=0.715
Treatment for HT	0.077 ± 0.076	=0.315	0.077 ± 0.076	=0.312	0.074 ± 0.076	=0.329	0.065 ± 0.074	=0.383
Mean arterial pressure	0.030 ± 0.080	=0.706	0.020 ± 0.080	=0.806	—		—	—
Heart rate	0.055 ± 0.080	=0.494	0.065 ± 0.080	=0.415	0.056 ± 0.076	=0.474	0.124 ± 0.078	=0.113
Metabolic Factors								
Ln Total/HDL cholesterol	—	—	0.097 ± 0.078	=0.215	—	—	—	—
Hemodynamic factors								
Central arterial SBP	—	—	—	—	0.065 ± 0.078	=0.403	—	—
Pb	—	—	—	—	—	—	**0.246** ± **0.075**	=**0.0013**
**Model r**^**2**^	**0.0947**	**<0.0001**	**0.1028**	**<0.0001**	**0.0976**	**<0.0001**	**0.1504**	**<0.0001**

IMT versus	Stand. β±sem	p-value	Partial r	(95% CI)
	Final model		Final model	
Age	**0.224** ± **0.081**	=**0.0064**	**0.207**	**(0.059 – 0.345)**
Sex (male)	0.131 ± 0.081	=0.118	0.120	(−0.031 – 0.264)
BMI	−0.023 ± 0.083	=0.783	−0.021	(−0.170 – 0.129)
Regular smoking	0.012 ± 0.081	=0.877	0.012	(−0.138 – 0.161)
Regular alcohol	−0.045 ± 0.080	=0.577	−0.043	(−0.191 – 0.108)
Diabetes mellitus	0.028 ± 0.072	=0.696	0.030	(−0.120 – 0.179)
Treatment for HT	0.064 ± 0.074	=0.384	0.067	(−0.084 – 0.214)
Mean arterial pressure	—	—	—	—
Heart rate	0.133 ± 0.078	=0.089	0.130	(−0.020 – 0.274)
Metabolic Factors				
Ln Total/HDL cholesterol	0.103 ± 0.075	=0.172	0.104	(−0.046 – 0.250)
Hemodynamic factors				
Central arterial SBP	—	—		
Pb	**0.248** ± **0.075**	=**0.0011**	0.246	(0.099 – 0.381)
**Model r**^**2**^	**0.1569**	**<0.0001**		

The basic model included age, sex, BMI, regular smoking, regular drinking, diabetes mellitus, treatment for hypertension, mean arterial pressure (except for models with hemodynamic pressure factors due to collinearity) and heart rate. Subsequent models included those metabolic or hemodynamic factors that were significant in bivariate associations (Table 2). The final model included both metabolic and hemodynamic factors, where the most significant for each were chosen, and total /HDL cholesterol was included as a comparator. Significant associations are shown in bold type. Β, slope.

*BMI* body mass index, *BP* blood pressure, *BSA* body surface area, *CI* confidence interval, *HDL* high-density lipoprotein, *HT* hypertension, *Ln*, natural logarithm, *Pb* backward wave pressure, *r*=Pearson’s correlation coefficient, *SBP* systolic blood pressure, Stand, standardised.

In middle-aged adults, prior to the inclusion of hemodynamic or metabolic factors in the model, age, BMI, MAP and HR were associated with carotid IMT independent of confounders (Table 4). Age, BMI and HR remained associated with carotid IMT independent of both hemodynamic and metabolic factors (Table 4). In separate models, brachial systolic BP, brachial pulse pressure, central arterial systolic BP, central arterial pulse pressure, Pb and peak P_QxZc_; but not blood glucose, triglycerides, and total/HDL cholesterol were associated with carotid IMT independent of confounders (Table 4). Central arterial systolic BP was the hemodynamic factor with the numerically greatest standardised beta value and lowest p-value. Hence, central arterial systolic BP was included in the final model. Although, not significantly associated with carotid IMT independent of confounders, total/HDL cholesterol had a numerically greater standardised beta value than triglycerides, and hence was included in the final model. When included in the same model, central arterial systolic BP, but not total/HDL cholesterol, was independently associated with carotid IMT (Table 4).

**Table 4:** Multivariate models showing associations between cardiovascular risk factors and carotid intima-media thickness (IMT) in middle-aged adults (age 35–59 years, n = 231).

IMT versus	Stand. β±sem	p-value	Stand. β±sem	p-value	Stand. β±sem	p-value	Stand. β±sem	p-value
	Model 1		Model 2		Model 3		Model 4	
Age	**0.284** ± **0.064**	< **0.0001**	**0.250** ± **0.070**	=**0.0004**	**0.278** ± **0.066**	< **0.0001**	**0.274** ± **0.066**	< **0.0001**
Sex (male)	0.136 ± 0.072	=0.062	0.134 ± 0.075	=0.076	0.127 ± 0.076	=0.098	0.121 ± 0.075	=0.106
BMI	**0.210** ± **0.071**	=**0.0034**	**0.208** ± **0.075**	=**0.0061**	**0.207** ± **0.072**	=**0.0044**	**0.199** ± **0.073**	=**0.0068**
Regular smoking	0.050 ± 0.065	=0.446	0.056 ± 0.074	=0.451	0.047 ± 0.066	=0.481	0.050 ± 0.065	=0.444
Regular alcohol	−0.034 ± 0.065	=0.606	−0.031 ± 0.069	=0.658	−0.035 ± 0.066	=0.595	−0.032 ± 0.065	=0.629
Diabetes mellitus	0.048 ± 0.064	=0.455	—	—	0.046 ± 0.065	=0.478	0.043 ± 0.065	=0.513
Treatment for HT	−0.088 ± 0.066	=0.181	−0.098 ± 0.067	=0.149	−0.089 ± 0.066	=0.177	−0.085 ± 0.066	=0.197
Mean arterial pressure	**0.168** ± **0.064**	=**0.0089**	**0.171** ± **0.068**	=**0.0123**	**0.168** ± **0.064**	=**0.0089**	**0.171** ± **0.064**	=**0.0081**
Heart rate	**0.153** ± **0.063**	=**0.0161**	**0.165** ± **0.065**	=**0.0123**	**0.150** ± **0.063**	=**0.0186**	**0.155** ± **0.063**	=**0.0151**
Metabolic Factors								
Ln glucose	—	—	0.086 ± 0.067	=0.202	—	—	—	—
Ln Triglycerides	—	—	—	—	0.026 ± 0.068	=0.698	—	—
Ln Total/HDL cholesterol	—	—	—	—	—	—	0.049 ± 0.065	=0.452
Hemodynamic factors								
Brachial systolic BP	—	—	—	—	—	—	—	—
Brachial pulse pressure	—	—	—	—	—	—	—	—
Central arterial SBP	—	—	—	—	—	—	—	—
Central arterial PP	—	—	—	—	—	—	—	—
Pb	—	—	—	—	—	—	—	—
Peak P_QxZc_	—	—	—	—	—	—	—	—
**Model r**^**2**^	**0.2218**	**<0.0001**	**0.2152**	**<0.0001**	**0.2224**	**<0.0001**	**0.2238**	**<0.0001**

IMT versus	Stand. β±sem	p-value	Stand. β±sem	p-value	Stand. β±sem	p-value	Stand. β±sem	p-value
	Model 5		Model 6		Model 7		Model 8	
Age	**0.264** ± **0.066**	< **0.0001**	**0.268** ± **0.066**	< **0.0001**	**0.262** ± **0.065**	< **0.0001**	**0.257** ± **0.066**	=**0.0001**
Sex (male)	0.132 ± 0.072	=0.071	**0.149** ± **0.072**	=**0.0390**	0.141 ± 0.072	=0.051	**0.167** ± **0.072**	=**0.0207**
BMI	**0.213** ± **0.071**	=**0.0028**	**0.218** ± **0.071**	=**0.0022**	**0.216** ± **0.070**	=**0.0025**	**0.228** ± **0.070**	=**0.0013**
Regular smoking	0.050 ± 0.065	=0.447	0.062 ± 0.065	=0.343	0.048 ± 0.065	=0.464	0.056 ± 0.065	=0.392
Regular alcohol	−0.033 ± 0.065	=0.614	−0.044 ± 0.065	=0.505	−0.034 ± 0.065	=0.600	−0.043 ± 0.065	=0.510
Diabetes mellitus	0.047 ± 0.064	=0.469	0.036 ± 0.064	=0.578	0.048 ± 0.064	=0.453	0.037 ± 0.064	=0.565
Treatment for HT	−0.094 ± 0.066	=0.153	−0.086 ± 0.066	=0.190	−0.096 ± 0.066	=0.146	−0.093 ± 0.066	=0.157
Mean arterial pressure	—	—	—	—	—	—	—	—
Heart rate	**0.153** ± **0.063**	=**0.0159**	**0.142** ± **0.065**	=**0.0248**	**0.171** ± **0.063**	=**0.0076**	**0.180** ± **0.064**	=**0.0053**
Metabolic Factors								
Ln glucose	—	—	—	—	—	—	—	—
Ln Triglycerides	—	—	—	—	—	—	—	—
Ln Total/HDL cholesterol	—	—	—	—	—	—	—	—
Hemodynamic factors								
Brachial systolic BP	**0.187** ± **0.065**	=**0.0046**	—	—	—	—	—	—
Brachial pulse pressure	—	—	**0.168** ± **0.063**	=**0.0098**	—	—	—	—
Central arterial SBP	—	—	—	—	**0.194** ± **0.065**	=**0.0033**	—	—
Central arterial PP	—	—	—	—	—	—	**0.189** ± **0.066**	=**0.0047**
Pb	—	—	—	—	—	—	—	—
Peak P_QxZc_	—	—	—	—	—	—	—	—
**Model r**^**2**^	**0.2260**	**<0.0001**	**0.2317**	**<0.0001**	**0.2282**	**<0.0001**	**0.2258**	**<0.0001**

IMT versus	Stand. β±sem	p-value	Stand. β±sem	p-value	Stand. β±sem	p-value	Partial r	(95% CI)
	Model 9		Model 10		Final model		Final model	
Age	**0.257** ± **0.068**	=**0.0002**	**0.273** ± **0.067**	< **0.0001**	**0.250** ± **0.067**	=**0.0002**	**0.244**	**(0.115 – 0.363)**
Sex (male)	**0.174** ± **0.072**	=**0.0170**	**0.144** ± **0.073**	=**0.0493**	0.125 ± 0.074	=0.093	0.113	(−0.019–0.241)
BMI	**0.244** ± **0.070**	=**0.0006**	**0.228** ± **0.071**	=**0.0015**	**0.202** ± **0.072**	=**0.0054**	**0.186**	**(0.055 – 0.310)**
Regular smoking	0.057 ± 0.065	=0.381	0.064 ± 0.065	=0.326	0.048 ± 0.065	=0.462	0.050	(−0.083 – 0.180)
Regular alcohol	−0.028 ± 0.065	=0.671	−0.025 ± 0.066	=0.701	−0.032 ± 0.065	=0.625	−0.033	(−0.164 – 0.099)
Diabetes mellitus	0.036 ± 0.064	=0.579	0.031 ± 0.065	=0.627	0.042 ± 0.064	=0.517	0.044	(−0.088 – 0.174)
Treatment for HT	−0.075 ± 0.065	=0.254	−0.076 ± 0.066	=0.250	−0.093 ± 0.066	=0.161	−0.094	(−0.223 – 0.038)
Mean arterial pressure	—	—	—	—	—	—	—	—
Heart rate	**0.177** ± **0.065**	=**0.0068**	0.145 ± 0.063	=**0.0225**	**0.173** ± **0.063**	=**0.0069**	**0.181**	**(0.050 – 0.305)**
Metabolic Factors								
Ln glucose	—	—	—	—	—	—	—	—
Ln Triglycerides	—	—	—	—	—	—	—	—
Ln Total/HDL cholesterol	—	—	—	—	0.055 ± 0.065	=0.400	0.057	(−0.076 – 0.187)
Hemodynamic factors								
Brachial systolic BP	—	—	—	—	—	—	—	—
Brachial pulse pressure	—	—	—	—	—	—	—	—
Central arterial SBP	—	—	—	—	**0.198** ± **0.066**	=**0.0028**	**0.200**	**(0.070 – 0.323)**
Central arterial PP	—	—	—	—	—	—	—	—
Pb	**0.152** ± **0.066**	=**0.0231**	—	—	—	—	—	—
Peak P_QxZc_	—	—	**0.135** ± **0.065**	=**0.0390**	—	—	—	—
**Model r**^**2**^	**0.2158**	**<0.0001**	**0.2126**	**<0.0001**	**0.2307**	**<0.0001**		

The basic model included age, sex, BMI, regular smoking, regular drinking, diabetes mellitus (except for models with glucose due to collinearity), treatment for hypertension, mean arterial pressure (except for models with hemodynamic pressure factors due to collinearity) and heart rate. Subsequent models included those metabolic or hemodynamic factors that were significant in bivariate associations (Table 2). The final model included both metabolic and hemodynamic factors, where the most significant for each were chosen, and total /HDL cholesterol was included as a comparator. Significant p-values are shown in bold type.

Β, slope, *BMI* body mass index, *BP* blood pressure, *BSA* body surface area, *CI* confidence interval, *HDL* high-density lipoprotein. *HT* hypertension, *Ln* natural logarithm, *Pb* backward wave pressure, Peak P_QxZc_, component of forward wave pressure generated by the product of peak Q and Zc, *PP* pulse pressure, r Pearson’s correlation coefficient, *SBP* systolic blood pressure; Stand, standardised.

In older adults, prior to the inclusion of hemodynamic or metabolic factors in the model, age and MAP were associated with carotid IMT independent of confounders (Table 5). However, age was not associated with carotid IMT independent of both hemodynamic and metabolic factors (Table 5). Male sex and HR were associated with carotid IMT independent of both hemodynamic and metabolic factors and confounders (Table 5). In separate models, brachial systolic BP, brachial pulse pressure, central arterial systolic BP, central arterial pulse pressure, Zc, Pb, and peak P_QxZc_, but not total/HDL cholesterol were associated with carotid IMT independent of confounders (Table 5). Pb was the hemodynamic factor with the numerically highest standardised beta value and lowest p-value. Hence, Pb was the hemodynamic factor included in the final model. When included in the same model Pb, but not total/HDL cholesterol, was associated with carotid IMT in older adults (Table 5).

**Table 5:** Multivariate models showing associations between cardiovascular risk factors and carotid intima-media thickness (IMT) in older adults (age ≥ 60 years, n=161).

IMT versus	Stand. β±sem	p-value	Stand. β±sem	p-value	Stand. β±sem	p-value	Stand. β±sem	p-value
	Model 1		Model 2		Model 3		Model 4	
Age	**0.166** ± **0.081**	=**0.0419**	0.175 ± 0.084	=**0.0375**	0.129 ± 0.082	=0.115	0.082 ± 0.084	=0.330
Sex (male)	0.170 ± 0.099	=0.089	0.165 ± 0.100	=0.101	0.177 ± 0.098	=0.074	0.168 ± 0.097	=0.084
BMI	0.034 ± 0.098	=0.727	0.036 ± 0.098	=0.712	0.013 ± 0.097	=0.890	−0.030 ± 0.097	=0.756
Regular smoking	0.058 ± 0.091	=0.524	0.059 ± 0.091	=0.522	0.061 ± 0.090	=0.496	0.031 ± 0.089	=0.732
Regular alcohol	−0.018 ± 0.085	=0.834	−0.014 ± 0.085	=0.872	−0.031 ± 0.084	=0.708	−0.046 ± 0.083	=0.576
Diabetes mellitus	−0.041 ± 0.082	=0.621	−0.047 ± 0.083	=0.571	−0.048 ± 0.080	=0.548	−0.051 ± 0.079	=0.519
Treatment for HT	0.021 ± 0.083	=0.802	0.016 ± 0.084	=0.849	0.031 ± 0.081	=0.704	0.043 ± 0.080	=0.589
Mean arterial pressure	**0.166** ± **0.079**	=**0.0381**	**0.159** ± **0.081**	=**0.0498**	—	—	—	—
Heart rate	0.079 ± 0.080	=0.327	0.070 ± 0.082	=0.390	0.105 ± 0.079	=0.185	0.133 ± 0.079	=0.094
Metabolic Factors								
Ln Total/HDL cholesterol	—	—	0.039 ± 0.084	=0.643	—	—	—	—
Hemodynamic factors								
Brachial systolic BP	—	—	—	—	**0.238** ± **0.080**	=**0.0033**	—	—
Brachial pulse pressure	—	—	—	—	—	—	**0.296** ± **0.082**	=**0.0004**
Central arterial SBP	—	—	—	—	—	—	—	—
Central arterial PP	—	—	—	—	—	—	—	—
Zc	—	—	—	—	—	—	—	—
Pb	—	—	—	—	—	—	—	—
Peak P_QxZc_	—	—	—	—	—	—	—	—
**Model r**^**2**^	**0.1045**	< **0.0001**	**0.1058**	**<0.0001**	**0.1301**	**<0.0001**	**0.1513**	**<0.0001**

IMT versus	Stand. β±sem	p-value	Stand. β±sem	p-value	Stand. β±sem	p-value	Stand. β±sem	p-value
	Model 5		Model 6		Model 7		Model 8	
Age	0.134 ± 0.081	=0.101	0.085 ± 0.082	=0.304	**0.162** ± **0.079**	=**0.0429**	0.078 ± 0.080	=0.332
Sex (male)	0.186 ± 0.098	=0.060	0.186 ± 0.096	=0.055	0.193 ± 0.098	=0.051	**0.212** ± **0.095**	=**0.0279**
BMI	0.020 ± 0.096	=0.838	−0.023 ± 0.096	=0.811	0.022 ± 0.096	=0.815	0.005 ± 0.093	=0.960
Regular smoking	0.053 ± 0.090	=0.557	0.010 ± 0.089	=0.913	0.053 ± 0.089	=0.553	0.024 ± 0.087	=0.780
Regular alcohol	−0.029 ± 0.084	=0.727	−0.045 ± 0.082	=0.584	−0.037 ± 0.083	=0.655	−0.039 ± 0.081	=0.631
Diabetes mellitus	−0.041 ± 0.080	=0.614	−0.035 ± 0.079	=0.658	−0.060 ± 0.081	=0.461	−0.074 ± 0.077	=0.339
Treatment for HT	0.026 ± 0.081	=0.746	0.030 ± 0.080	=0.711	0.058 ± 0.082	=0.486	0.035 ± 0.079	=0.660
Mean arterial pressure	—	—	—	—	0.112 ± 0.080	=0.167	—	—
Heart rate	0.122 ± 0.080	=0.130	**0.170** ± **0.080**	=**0.0361**	0.078 ± 0.078	=0.318	**0.180** ± **0.079**	=**0.0246**
Metabolic Factors								
Ln Total/HDL cholesterol	—	—	—	—	—	—	—	—
Hemodynamic factors								
Brachial systolic BP		—	—	—	—	—	—	—
Brachial pulse pressure	—	—	—	—	—	—	—	—
Central arterial SBP	**0.242** ± **0.080**	=**0.0028**	—	—	—	—	—	—
Central arterial PP		—	—	**0.322** ± **0.083**	=**0.0002**	—	—	—
Zc	—	—	—	—	**0.212** ± **0.080**	=**0.0087**	—	—
Pb	—	—	—	—	—	—	**0.358** ± **0.080**	< **0.0001**
Peak P_QxZc_	—	—	—	—	—	—	—	—
**Model r**^**2**^	**0.1315**	< **0.0001**	**0.1624**	**<0.0001**	**0.1448**	**<0.0001**	**0.1869**	**<0.0001**

IMT versus	Stand. β±sem	p-value	Stand. β±sem	p-value	Partial r	(95%CI)
	Model 9		Final model		Final model	
Age	0.097 ± 0.081	=0.232	0.091 ± 0.082	=0.271	0.090	(−0.070 – 0.245)
Sex (male)	0.163 ± 0.096	=0.093	**0.205** ± **0.096**	=**0.0339**	**0.172**	**(0.013 – 0.322)**
BMI	−0.024 ± 0.096	=0.799	0.007 ± 0.093	=0.939	0.006	(−0.153 – 0.165)
Regular smoking	0.045 ± 0.088	=0.609	0.025 ± 0.087	=0.775	0.023	(−0.136 – 0.182)
Regular alcohol	−0.043 ± 0.082	=0.600	−0.033 ± 0.081	=0.682	−0.033	(−0.191 – 0.126)
Diabetes mellitus	−0.084 ± 0.079	=0.284 −0.082 ± 0.078	=0.294 −0.085	(−0.241 – 0.075)		
Treatment for HT	0.033 ± 0.080	=0.679	0.027 ± 0.080	=0.735	0.028	(−0.132 – 0.186)
Mean arterial pressure	—	—	—	—	—	—
Heart rate	0.118 ± 0.078	=0.130	**0.167** ± **0.081**	=**0.0406**	**0.166**	**(**−**0.007 – 0.317)**
Metabolic Factors						
Ln Total/HDL cholesterol	—	—	0.057 ± 0.079	=0.471	0.059	(−0.101 – 0.216)
Hemodynamic factors						
Brachial systolic BP		—	—	—	—	—
Brachial pulse pressure	—	—	—	—	—	—
Central arterial SBP		—	—	—	—	—
Central arterial PP		—	—	—	—	—
Zc	—	—	—	—	—	—
Pb	—	—	**0.356** ± **0.080**	< **0.0001**	**0.342**	**(0.192 – 0.474)**
Peak P_QxZc_	**0.304** ± **0.079**	=**0.0002**	—	—	—	—
**Model r**^**2**^	**0.1605**	< **0.0001**	**0.1897**	**<0.0001**		

The basic model included age, sex, BMI, regular smoking, regular drinking, diabetes mellitus, treatment for hypertension, mean arterial pressure (except for models with hemodynamic pressure factors due to collinearity) and heart rate. Subsequent models included those metabolic or hemodynamic factors that were significant in bivariate associations (Table 2). The final model included both metabolic and hemodynamic factors, where the most significant for each were chosen, and total /HDL cholesterol was included as a comparator. Significant p-values are shown in bold type. Β, slope.

*BMI* body mass index, *BP* blood pressure, *BSA* body surface area, *CI* confidence interval, *HDL* high-density lipoprotein, *HT* hypertension, *Ln* natural logarithm, *Pb* backward wave pressure, Peak P_QxZc_, component of forward wave pressure generated by the product of peak Q and Zc, *PP* pulse pressure; r=Pearson’s correlation coefficient; SBP, systolic blood pressure; Stand, standardised; Zc, aortic characteristic impedance.

### Hemodynamic and metabolic factors and odds of increased carotid IMT independent of confounders in different age groups

**Fig. 1:**
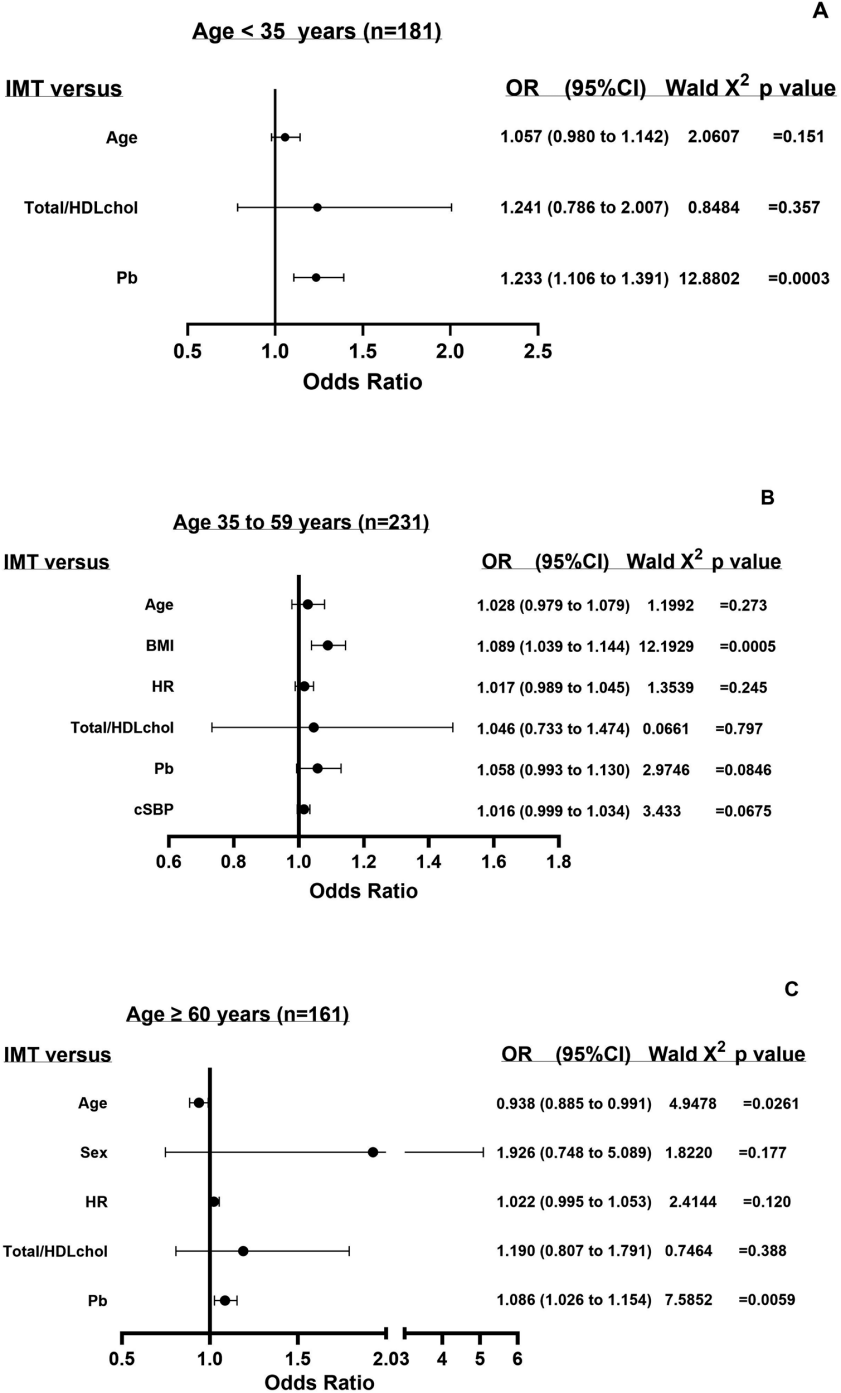
The impact of hemodynamic and metabolic cardiovascular risk factors on the odds of increased carotid intima media thickness in the three age groups. The panels show the odds ratios of an increased carotid intima media thickness associated with one unit increase in hemodynamic and metabolic cardiovascular risk factors in young (age 35 years, **A**), middle-aged (age 35 to 59 years, **B**), and older adults (age ≥ 60 years, **C**). The models included both metabolic and hemodynamic factors (cSBP and Pb separately), where the most significant for each were chosen, and age and total/HDL cholesterol were included as comparators (final model as in Tables 3, 4 and 5). Also included in the models were sex, BMI, regular smoking, regular drinking, diabetes mellitus, treatment for hypertension, and heart rate. BMI body mass index, CI confidence interval, cSBP central arterial systolic blood pressure; HR, heart rate, IMT carotid intima-media thickness, OR odds ratio, Pb backward wave pressure, Total/HDLchol Total to HDL cholesterol ratio.

Figure 1 shows the impact of hemodynamic and metabolic cardiovascular risk factors on the odds of increased carotid IMT independent of confounders in the three age groups. In young adults, one unit increase in Pb, but neither age nor total/HDL cholesterol was associated with an increased carotid IMT (Fig. 1A). In middle-aged adults, one unit increase in BMI was the only cardiovascular risk factor significantly associated with an increased carotid IMT (Fig. 1B). In older adults, one unit increase in Pb and age, but not total/HDL cholesterol, sex or HR were associated with an increased carotid IMT (Fig. 1C).

### Performance of hemodynamic and metabolic factors in identifying individuals with increased carotid IMT in different age groups

**Fig. 2:**
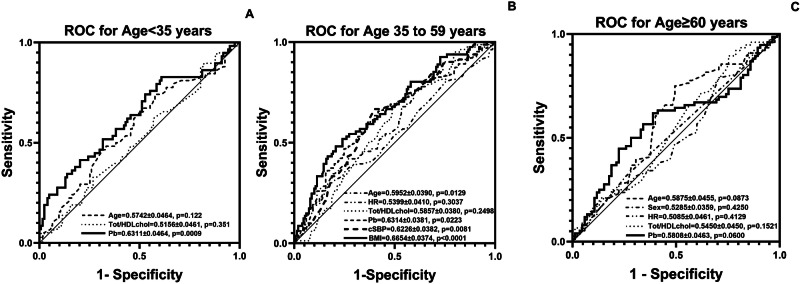
Performance of hemodynamic and metabolic factors in identifying individuals with increased carotid IMT in different age groups. The figure shows the area under the receiver operator characteristic (ROC) curves showing the performance of individual hemodynamic or metabolic factors in identifying individuals with increased carotid IMT in young (age <35 years, **A**), middle-aged (age 35 to 59 years, **B**), and older adults (age ≥ 60 years, **C**). BMI body mass index, cSBP central arterial systolic blood pressure, HR heart rate, Pb backward wave pressure, Tot/HDLchol Total to HDL cholesterol ratio.

Figure 2 shows the receiver operator characteristic (ROC) curve analysis of individual hemodynamic and metabolic cardiovascular factors in identifying individuals with increased carotid IMT in the three different age groups. In young adults (Fig. 2A), only Pb significantly identified individuals with increased carotid IMT. In middle-aged adults (Fig. 2B), BMI, central arterial systolic BP, Pb, and age significantly identified individuals with increased carotid IMT. In older adults (Fig. 2C), age and Pb showed a trend to identify individuals with increased carotid IMT.

The impact of adding hemodynamic or metabolic factors to the base model, consisting of conventional risk factors, on predictive performance for an increased carotid IMT is shown in Table 6 for all participants as well as in young, middle-aged and older adults. The impact of adding age to the base model (excluding age) is included for comparison. In all participants and in young adults, Pb was the only factor to show a significant improvement in predictive performance for detecting an increased carotid IMT. In middle-aged adults, significant improvements in predictive performance for detecting an increased carotid IMT were shown with BMI, and Pb showed only weak significant improvements. In older adults, age and Pb showed significant improvements in predictive performance.

**Table 6:** Impact of adding hemodynamic or metabolic factors to the base model, consisting of conventional risk factors for an increased carotid IMT, on predictive performance for an increased carotid IMT (Incr. IMT), in all participants as well as in young, middle-aged and older adults.

	AUC and difference				Integrated discrimination improvement			Net reclassification improvement		
Incr. IMT vs Normal	Base	Added	Δ	(SE)	IDI	(95% CI)	P	NRI	(95% CI)	P
Factor										
All participants										
Age	0.610	0.618	0.008	(0.007)	0.003	(−0.001 to 0.007)	=0.186	0.046	(−0.123 to 0.214)	=0.597
Tot/HDLchol	0.618	0.620	0.002	(0.006)	0.003	(−0.002 to 0.007)	=0.224	0.020	(−0.147 to 0.187)	=0.819
Pb	0.618	0.667	0.049	(0.017)**	**0.033**	**(0.018 to 0.048)**	< **0.0001**	**0.425**	**(0.260 to 0.591)**	< **0.0001**
Young adults										
Age	0.560	0.597	0.037	(0.040)	0.015	(−0.003 to 0.033)	=0.100	0.262	(−0.047 to 0.571)	=0.100
Tot/HDLchol	0.597	0.585	−0.012	(0.012)	0.003	(−0.007 to 0.013)	=0.517	0.092	(−0.217 to 0.400)	=0.564
Pb	0.597	0.684	0.087	(0.045)*	**0.083**	**(0.036 to 0.131)**	=**0.0006**	**0.469**	**(0.165 to 0.774)**	=**0.0032**
Middle-aged adults										
Age	0.680	0.695	0.015	(0.015)	0.012	(−0.003 to 0.026)	=0.122	0.128	(−0.141 to 0.398)	=0.352
BMI	0.622	0.695	0.073	(0.034)*	**0.065**	**(0.031 to 0.099)**	=**0.0002**	**0.492**	**(0.229 to 0.754)**	=**0.0004**
Tot/HDLchol	0.695	0.694	−0.001	(0.001)	−0.001 (−0.0003 to 0.0003)	=0.970	−0.052 (−0.318 to 0.214)	=0.707		
Pb^†^	0.695	0.713	0.018	(0.013)	**0.014**	**(0.001 to 0.028)**	=**0.042**	**0.275**	**(0.007 to 0.543)**	=**0.046**
cSBP^†^	0.695	0.706	0.011	(0.014)	**0.016**	**(0.001 to 0.032)**	=**0.041**	0.125	(−0.143 to 0.393)	=0.365
Older adults										
Age	0.601	0.631	0.030	(0.032)	0.019	(−0.002 to 0.039)	=0.074	**0.503**	**(0.209 to 0.797)**	=**0.0014**
Tot/HDLchol	0.631	0.632	0.001	(0.015)	0.005	(−0.006 to 0.016)	=0.386	−0.012	(−0.321 to 0.298)	=0.941
Pb	0.631	0.681	0.050	(0.037)	**0.048**	**(0.015 to 0.082)**	=**0.0050**	**0.467**	**(0.170 to 0.765)**	= **0.0031**

The impact of adding age to the base model is included for comparison.

The base model for Tot/HDLchol, Pb and cSBP included age, sex, BMI, regular smoking, regular alcohol, diabetes mellitus, treatment for hypertension, and heart rate. For age the base model excluded age and for BMI the base model excluded BMI. IDI and NRI estimates are given with 95% CI. Significant p-values are shown in bold type. Tot/HDLchol, total to HDL cholesterol ratio; Pb, backward wave pressure.

*cSBP* central arterial systolic blood pressure, *BMI* body mass index, *IDI* integrated discrimination improvement, *NRI* net reclassification improvement, *AUC* area under the receiver operating characteristic (ROC) curve.

*p < 0.05.

**p < 0.005 for significant change in AUC.

^†^Pb and cSBP are in separate models.

## Discussion

In a community sample of adults of African ancestry living in Sub-Saharan Africa, we show that hemodynamic, and not metabolic cardiovascular risk factors are associated with carotid IMT. In addition, we showed that the primary hemodynamic and metabolic cardiovascular risk factors associated with carotid IMT differ across adult age groups. In young adults, hemodynamic factors (Pb) primarily impacted on carotid IMT. In older adults, Pb and age were associated carotid IMT; whereas in middle-aged adults, BMI, central arterial systolic BP and Pb were the primary factors associated with carotid IMT. Blood flow (SV and Q) and smoking status were not related to carotid IMT at any adult age. Moreover, plasma lipid concentrations were not associated with carotid IMT at any adult age. Only Pb was associated with increased carotid IMT in young adults; whereas in older adults age and Pb were determinants of increased carotid IMT, and in middle-aged adults, BMI was the primary determinant of increased IMT. Furthermore, significant improvements in predictive performance for detecting an increased carotid IMT were shown with Pb (young and older adults), and BMI (middle-aged adults). Hence, hemodynamic factors and not plasma lipid concentrations are the predominant cardiovascular risk factors associated with carotid IMT throughout the adult lifespan in individuals of African ancestry, and BMI only has an impact during middle-age.

To our knowledge, our study is the first to report on the hemodynamic and metabolic cardiovascular risk factors associated with carotid IMT in young adults of African ancestry living in Sub-Saharan Africa. Furthermore, our study is the first to report that the primary hemodynamic and metabolic cardiovascular risk factors associated with carotid IMT differ with age in a group of African ancestry. Although a large study in four Sub-Saharan African countries (Burkina Faso, Kenya, South Africa, Ghana) identified factors associated with carotid IMT, these data were limited to adults aged 40–60 years [10]. In this study, brachial systolic BP, but not LDL cholesterol, was associated with carotid IMT independent of confounders. Nevertheless, previous data assessing the cardiovascular risk factors associated with carotid IMT in young adults of African ancestry are lacking. In other populations (Chinese [11] and European [12]) the determinants of carotid IMT have been shown to differ according to age. In Chinese, the major determinants of carotid IMT beyond age and gender, were BMI at age 35–44 years, LDL cholesterol and brachial systolic BP at age 45–54 years, and brachial systolic BP at 55–64, 65–74 and aged 75 years or older [11]. In Europeans, aged 24 to 39 years, systolic BP, but neither LDL nor HDL cholesterol were independently related to carotid IMT [12]. However, in the same Europeans studied 6 years later, carotid IMT was associated with both brachial systolic BP and HDL cholesterol (inversely) [12]. Although data in adults aged<35 years are limited, it appears from the present study and a study in Europeans [12], that hemodynamic factors (Pb and brachial systolic BP respectively) are more important than metabolic factors (blood lipids) in young adults. As adults mature into middle-age, BMI (present study) and metabolic factors (HDL cholesterol in Europeans [12], LDL cholesterol in Chinese [11] seem to play a role in addition to hemodynamic factors. However, beyond middle-age, hemodynamic factors appear to predominate over metabolic factors [11] and present study.

Reasons for the differential impact of cardiovascular risk factors on carotid IMT with age are unclear. Nevertheless, hemodynamic factors consistently affect carotid IMT throughout the adult age range [11, 12, 23] and present study. On assessing the impact of hemodynamic factors, brachial pulse pressure in comparison to systolic, diastolic, and mean BP, may be the best predictor of carotid IMT in middle-aged (≥45 years) [24] and older (≥65 years) [24, 25] adults. Indeed, elevated brachial pulse pressure is associated with the progression of carotid IMT in older adults [26]. Moreover, increases in central arterial systolic BP, but not peripheral systolic BP, are associated with increases in carotid IMT in middle-aged and older adults [27]. Similarly, we show that central arterial pressures are associated with carotid IMT in middle-aged adults of African ancestry, and that in older adults central arterial pressures were numerically stronger than peripheral BP in association with carotid IMT. Moreover, we extend these findings to show that in young adults of African ancestry carotid IMT is associated with central arterial pressures (Pb) but not peripheral BP. The predominant effect of central arterial pressures on carotid IMT in young adults of African ancestry may be attributed to the high central arterial pressures and stiffness reported in young African adults [8]. Indeed, central arterial stiffness, central arterial pressure [8] and central arterial pressure augmentation [9] are elevated in African compared with European individuals, particularly among young adults [8].

Although, the role of wave reflection in cardiovascular disease is increasingly documented [28–30], our findings of a consistent association of central arterial pressures with carotid IMT in all adult age groups may be a consequence of vascular changes rather than a cause of increases in carotid IMT. In this regard, although intima-media thickening represents vascular remodelling and consequent decreases in lumen diameter in response to increases in BP [31], arterial narrowing in turn increases wave reflection and subsequently central arterial SBP and PP [28].

The predominant effect of hemodynamic rather than metabolic risk factors on carotid IMT in people of African ancestry is not surprising. In contrast to European populations, in Sub-Saharan African countries, hypertension, rather than dyslipidaemia, is the primary risk factor for CVD [7]. In this regard, in Sub-Saharan Africa, the prevalence of hypertension amongst adults is higher [5], whereas the prevalence of dyslipidaemia is lower [32], than in other regions of the world. Moreover, the proportion of individuals achieving BP control in Sub-Saharan Africa is lower than in other regions of the world [5]. Similarly, in Chinese populations, who also have lower cholesterol concentrations than European populations [33], hypertension has a higher impact on carotid IMT compared to dyslipidemia and diabetes [34].

Our data showing no relationships between lipid concentrations and carotid IMT independent of confounders in adults of African ancestry concur with findings reported in prior studies. In Nigerian Africans, BP was reported to be the strongest modifiable risk factor associated with carotid IMT [35]. In African populations, LDL cholesterol has a weaker negative impact on carotid IMT compared to in European populations [6]. Moreover, in four separate sub-Saharan African countries (Burkina Faso, Kenya, South Africa, Ghana), brachial systolic BP, but not LDL cholesterol, was associated with carotid IMT independent of confounders [10]. These data are supported by a recent systematic review which identified hypertension rather than dyslipidaemia, as the primary risk factor for CVD in Sub-Saharan African countries [7].

The lack of impact of blood lipids on carotid IMT in persons of African ancestry may be explained by the lower frequency of unfavourable lipid profiles (increased LDL cholesterol and triglycerides, and decreased HDL cholesterol) in Sub-Saharan Africa compared to Western Europe and North America [32]. Indeed, unfavourable lipid profiles are a major cause of atherosclerosis in communities of European ancestry [36]. Moreover, reductions in age-standardised death rates for ischemic stroke and ischemic heart disease in Europe between 1990 and 2019 have been attributed to decreases in total and LDL cholesterol concentrations [32].

The identification of a consistent association of hemodynamic factors with carotid IMT throughout the adult lifespan is of clinical relevance. In this regard, the high prevalence of hypertension, lack of awareness of hypertension and poor BP control in South Africans adults [37] is of concern. In particular, poor BP control and lack of awareness of hypertension seems to be particularly prevalent among young adults in South Africa [37]. As increased carotid IMT is associated with cardiovascular events (stroke, CLTI and CAD) in people of African ancestry living in Sub-Saharan African [15, 38–40], identification of factors that can be targeted to prevent increases in carotid IMT is paramount. In this regard, significant improvements in predictive performance for detecting an increased carotid IMT were shown with Pb in young and older adults. The present study therefore, highlights that in the prevention of cardiovascular disease it is imperative to screen for and manage high BP and hence Pb, throughout the adult lifespan. Moreover, screening for hypertension, managing hypertension and maintaining BP control are highly relevant to reduce cardiovascular risk in young adults of African ancestry.

There are several limitations to the present study. As the present study was cross-sectional in design, the relationships noted may not be cause and effect and may be attributed to residual confounding. Further studies evaluating the long-term impact of elevated BP on carotid IMT throughout the adult lifespan in individuals of African ancestry are therefore required. However, the present study provides the critical evidence in support of such future studies. Second, as more women than men participated in this study, the results may pertain more to women than to men. Notably, the high proportion of women compared to men was consistent across all three age groups. The strengths of our study include the thorough assessment of lipid profiles (total, LDL, and HDL cholesterol, total/HDL cholesterol ratio, and triglycerides) and hemodynamic factors (peripheral BP, central arterial pressures and components, and blood flow). Importantly, our data pertain to individuals of African ancestry living in Sub-Saharan Africa, and hence should not be extended to European or US cohorts.

## Conclusions

In a population of African ancestry living in Sub-Saharan Africa, hemodynamic factors but not blood lipids, are associated with carotid IMT throughout the adult age range. Furthermore, the cardiovascular risk factors associated with carotid IMT differ across age groups. Hemodynamic factors, primarily central arterial pressures predominate in young and older adults; whereas BMI is the main contributor in middle-age.

## Summary

### What is known about the topic

- Cardiovascular events occur 20 years earlier in individuals of African descent living in Sub-Saharan Africa compared to in individuals of European descent.
- Carotid artery intima-media thickness (IMT), an important screening tool for cardiovascular disease, is lower in African populations living in Sub-Saharan Africa compared to other population groups.
- The risk factors for cardiovascular disease differ between population groups and according to age.

### What this study adds

- In individuals of African descent living in South Africa:
- Lipids are not associated with carotid IMT at any age.
- Hemodynamic factors (primarily central arterial backward wave pressure) are associated with carotid IMT independent of confounders, particularly in young adults ( < 35years of age).
- BMI is independently associated with carotid IMT only in middle-aged adults (35–59 years of age).

## Supplementary information

Online Supplement

## Data Availability

All relevant data are contained within the manuscript.
