# Supplementary material for: Hemodynamic factors primarily impact on carotid IMT in young adults of African Ancestry in Sub-Saharan Africa
Source: J Hum Hypertens. 2026 Feb 7;40(4):265–80. doi: 10.1038/s41371-026-01119-8 (PMC13068520; doi:10.1038/s41371-026-01119-8)
Supplement: Supplementary file 1 — Online Supplement [file 41371_2026_1119_MOESM1_ESM.docx]

**On-line Supplement**

**Hemodynamic Factors Primarily Impact on Carotid IMT in Young Adults of African Ancestry in Sub-Saharan Africa.**

Nico Malan^1^, Gavin R Norton^1^, Vernice R Peterson^1^, Nonhlanhla H Mthembu^1^, Carlos D Libhaber^1^, Andrea Kolkenbeck-Ruh^1^, Grace Tade^1^, Pinhas Sareli^1^, Patrick H Dessein^1^, Angela J Woodiwiss^1^.

^1^From the Cardiovascular Pathophysiology and Genomics Research Unit, Department of Physiology, School of Biomedical Sciences, Faculty of Health Sciences, University of the Witwatersrand, Johannesburg, South Africa.

Running title: Age, risk factors and IMT.

This work was supported by the Circulatory Disorders Research Trust, the University Research Council of the University of the Witwatersrand, the South African National Research Foundation and The South African Medical Research Council.

NM, GRN, and AJW contributed equally to this work.

Correspondence and reprint requests: Angela J Woodiwiss, Cardiovascular Pathophysiology and Genomics Research Unit, Department of Physiology, School of Biomedical Sciences, University of the Witwatersrand Medical School, 7 York Road, Parktown, 2193, Johannesburg, South Africa. Tel + 27 11 717 2363, e-mail: [angela.woodiwiss@wits.ac.za](mailto:angela.woodiwiss@wits.ac.za)

**Table S1.** Characteristics of all study participants.

All

___________________________________________________________________

Sample size 573

% women (n) 69.3 (397)

% postmenopausal women (n) 48.4 (192)

Age (years) 47.0±17.8

Body mass index (kg/m^2^) 30.0±7.7

% Overweight (n) 25.0 (143)

% Obese (n) 46.4 (266)

% Hypertensive (n) 46.3 (265)

% Treated for hypertension (n) 27.6 (158)

% Hypertensives treated for hypertension (n) 59.6 (158/265)

% Uncontrolled BP (n) 34.2 (196)

% Hypertensives with uncontrolled BP (n) 74.0 (196/265)

% Regular smoking (n) 15.0 (86)

% Regular alcohol (n) 20.4 (117)

% Diabetes mellitus (n) 12.2 (70)

Carotid intima media thickness (IMT) (mm) 0.65±0.14

% Increased IMT (n) 37.5 (215)

% Plaque (n) 4.5 (26)

Metabolic Factors (Fasting plasma concentrations)

Glucose (mmol/l) 4.60 (4.20 to 5.10)

Glycated haemoglobin (%) 5.80 (5.50 to 6.10)

Insulin (μU/ml) 6.71 (4.29 to 12.33)

HOMA-IR 1.41 (0.86 to 2.82)

Total cholesterol (mmol/l) 4.63 (4.10 to 5.23)

LDL cholesterol (mmol/l) 2.70 (2.24 to 3.19)

HDL cholesterol (mmol/l) 1.40 (1.29 to 1.50)

Triglycerides (mmol/l) 1.10 (0.80 to 1.52)

Total/HDL cholesterol ratio 3.46 (2.88 to 3.85)

Hemodynamic factors

Brachial systolic BP (SBP) (mm Hg) 127±21

Brachial diastolic BP (mm Hg) 8212

Brachial pulse pressure (mm Hg) 44.2±14.1

Mean arterial pressure (MAP) (mm Hg) 98±15

Central arterial systolic BP (SBPc) (mm Hg) 118±21

Central arterial pulse pressure (PPc) (mm Hg) 34.7±13.2

Heart rate (beats/min) 67.5±12.3

Peak aortic flow (Q) (mls/sec) 345±191

Stroke volume (SV) (mls/beat) 79.5±38.0

Stroke volume (mls/beat.BSA) 44.3±20.9

Aortic characteristic impedance (Zc)(dynes.cm^-5^) 86.1±44.2

Total arterial compliance (TAC) (mm Hg/mls.bt) 2.49±1.31

Backward wave pressure (Pb) (mmHg) 12.9±5.7

Peak P_QxZc_ (mm Hg) 25.1±8.0

___________________________________________________________________

Data are shown as mean±SD, proportions, or median and interquartile range. BP, blood pressure; BSA, body surface area; HDL, high-density lipoprotein; HOMA-IR, homeostatic model assessment for insulin resistance; LDL, low-density lipoprotein; Peak P_QxZc_, component of forward wave pressure generated by the product of peak Q and Zc.

**Table S2.** Bivariate associations between cardiovascular risk factors and carotid intima-media thickness (IMT) in all participants (n=573).

**IMT versus** r (95% CI) p-value

___________________________________________________________________

Age **0.66 (0.61 – 0.71) <0.0001**

Sex (male) 0.02 (-0.06 – 0.10) =0.649

BMI **0.28 (0.21 – 0.36) <0.0001**

Hypertension **0.40 (0.33 – 0.47) <0.0001**

Regular smoking 0.02 (-0.06 – 0.10) =0.605

Regular alcohol 0.06 (-0.02 – 0.14) =0.151

Diabetes mellitus  **0.21 (0.13 – 0.29) <0.0001**

Treatment for HT **0.32 (0.25 – 0.39) <0.0001**

Metabolic Factors

Ln glucose **0.28 (0.20 – 0.35) <0.0001**

Ln glycated haemoglobin **0.21 (0.12 – 0.31) <0.0001**

Ln HOMA-IR **0.10 (0.01 – 0.18) =0.024**

Ln total cholesterol **0.29 (0.22 – 0.37) <0.0001**

Ln LDL cholesterol **0.30 (0.23 – 0.38) <0.0001**

Ln HDL cholesterol **-0.14 (-0.22 – -0.06) =0.0007**

Ln Triglycerides **0.36 (0.28 – 0.42) <0.0001**

Ln Total/HDL cholesterol **0.34 (0.26 – 0.41) <0.0001**

Hemodynamic factors

Brachial systolic BP **0.44 (0.37 – 0.51) <0.0001**

Brachial diastolic BP **0.25 (0.17 – 0.33) <0.0001**

Brachial pulse pressure **0.44 (0.38 – 0.51) <0.0001**

Mean arterial pressure **0.37 (0.30 – 0.44) <0.0001**

Central arterial SBP **0.46 (0.39 – 0.52)**  <**0.0001**

Central arterial PP **0.49 (0.43 – 0.55) <0.0001**

Heart rate **0.11 (0.03 – 0.19) =0.0067**

Peak aortic flow **0.15 (0.07 – 0.23) =0.0003**

Stroke volume **0.19 (0.11 – 0.27) <0.0001**

Stroke volume/BSA **0.15 (0.07 – 0.23) =0.0005**

Zc  **0.15 (0.06 – 0.22) =0.0005**

TAC  **-0.15 (-0.23 – -0.07) =0.0003**

Pb **0.47 (0.40 – 0.53)**  <**0.0001**

Peak P_QxZc_  **0.41 (0.34 –0.48)**  <**0.0001**

___________________________________________________________________

Significant associations are shown in bold type. BMI, body mass index; BP, blood pressure; BSA, body surface area; CI, confidence interval; HDL, high-density lipoprotein; HOMA-IR, homeostatic model assessment for insulin resistance; HT, hypertension; LDL, low-density lipoprotein; Ln, natural logarithm; Pb, backward wave pressure; PP, pulse pressure; Peak P_QxZc_, component of forward wave pressure generated by the product of peak Q and Zc; r=Pearson’s correlation coefficient; SBP, systolic blood pressure; TAC, total arterial compliance; Zc, aortic characteristic impedance.

**Table S3.** Multivariate models showing associations between cardiovascular risk factors and carotid intima-media thickness (IMT) in all participants (n=573).

**IMT versus** Stand. β±sem p-value Stand. β±sem p-value Stand. β±sem p-value Stand. β±sem p-value

________________________________________________________________________________________________________

Model 1 Model 2 Model 3 Model 4

Age **0.595±0.039** **<0.0001** **0.589±0.040** **<0.0001** **0.602±0.046** **<0.0001** **0.598±0.040** **<0.0001**

Sex (male) **0.098±0.037 =0.0087** **0.097±0.038 =0.0115** **0.107±0.037 =0.0246** **0.084±0.040 =0.0350**

BMI **0.079±0.038** **=0.0369 0.081±0.039** **=0.0408** 0.054±0.041 =0.287 0.076±0.041 =0.064

Regular smoking 0.039±0.035 =0.262 0.039±0.040 =0.287 0.027±0.036 =0.564 0.039±0.037 =0.288

Regular alcohol -0.030±0.034 =0.383 -0.028±0.035 =0.420 -0.009±0.036 =0.849 -0.039±0.036 =0.281

Diabetes mellitus 0.007±0.034 =0.844 — — — — — —Treatment for HT -0.012±0.037 =0.753 -0.017±0.038 =0.661 -0.012±0.043 =0.820 -0.031±0.039 =0.422

Mean arterial pressure **0.104±0.035** **=0.0028 0.107±0.036**  **=0.0028** 0.063±0.035 =0.168 **0.098±0.036** **=0.0073**

Heart rate **0.078±0.032** **=0.0155 0.080±0.033** **=0.0156 0.104±0.031** **=0.0118 0.067±0.034** **=0.0483**

Metabolic Factors

Ln glucose — — 0.009±0.035 =0.798 — — — —

Ln glycated hemoglobin — — — — -0.018±0.036 =0.701 — —

Ln HOMA-IR — — — — — — 0.008±0.034 =0.822

Ln total cholesterol — — — — — — — —

Ln LDL cholesterol — — — — — — — —

Ln HDL cholesterol — — — — — — — —

Ln Triglycerides — — — — — — — —

Ln Total/HDL cholesterol — — — — — — — —

Hemodynamic factors

Brachial systolic BP — — — — — — — —

Brachial diastolic BP — — — — — — — —

Brachial pulse pressure — — — — — — — —

Central arterial SBP — — — — — — — —

Central arterial PP — — — — — — — —

Peak aortic flow — — — — — — — —

Stroke volume — — — — — — — —

Stroke volume/BSA — — — — — — — —

Zc — — — — — — — —

TAC — — — — — — — —

Pb — — — — — — — —

Peak P_QxZc_ — — — — — — — —

**Model r^2^** **0.4639** **<0.0001 0.4600** **<0.0001** **0.4116** **<0.0001 0.4472** **<0.0001**

________________________________________________________________________________________________________

The basic model included age, sex, BMI, regular smoking, regular drinking, diabetes mellitus (except for models with glucose, glycated haemoglobin or HOMA-IR due to collinearity), treatment for hypertension, mean arterial pressure (except for models with hemodynamic pressure factors due to collinearity) and heart rate. Subsequent models included those metabolic or hemodynamic factors that were significant in bivariate associations (Table 2). The final model included both metabolic and hemodynamic factors, where the most significant for each were chosen, and total /HDL cholesterol was included as a comparator. Significant associations are shown in bold type. Β, slope; BMI, body mass index; BP, blood pressure; BSA, body surface area; CI, confidence interval; HDL, high-density lipoprotein; HOMA-IR, homeostatic model assessment for insulin resistance; HT, hypertension; LDL, low-density lipoprotein; Ln, natural logarithm; Pb, backward wave pressure; Peak P_QxZc_, component of forward wave pressure generated by the product of peak Q and Zc; PP, pulse pressure; r=Pearson’s correlation coefficient; SBP, systolic blood pressure; Stand, standardised; TAC, total arterial compliance; Zc, aortic characteristic impedance.

**Table S3 continued (1).** Multivariate models showing associations between cardiovascular risk factors and carotid intima-media thickness (IMT) in all participants (n=573).

**IMT versus** Stand. β±sem p-value Stand. β±sem p-value Stand. β±sem p-value Stand. β±sem p-value

________________________________________________________________________________________________________

Model 5 Model 6 Model 7 Model 8

Age **0.596±0.042** **<0.0001** **0.576±0.041** **<0.0001** **0.597±0.039** **<0.0001** **0.606±0.042** **<0.0001**

Sex (male) **0.097±0.038 =0.0105** **0.106±0.038 =0.0049** **0.079±0.039 =0.0420** **0.104±0.038 =0.0067**

BMI **0.079±0.038** **=0.0375 0.078±0.038** **=0.0412** 0.068±0.039 =0.078 **0.083±0.038 =0.0314**

Regular smoking 0.039±0.035 =0.263 0.038±0.035 =0.270 0.037±0.035 =0.289 0.041±0.035 =0.240

Regular alcohol -0.030±0.034 =0.384 -0.027±0.034 =0.417 -0.024±0.034 =0.488 -0.029±0.034 =0.393

Diabetes mellitus 0.007±0.034 =0.843 0.004±0.034 =0.906 -0.001±0.034 =0.983 0.009±0.034 =0.801 Treatment for HT -0.012±0.037 =0.754 -0.011±0.037 =0.768 -0.012±0.037 =0.745 -0.010±0.038 =0.790

Mean arterial pressure **0.105±0.035** **=0.0028 0.103±0.035**  **=0.0032 0.103±0.035 =0.0031 0.106±0.035** **=0.0026**

Heart rate **0.078±0.032** **=0.0158 0.081±0.032** **=0.0119 0.076±0.032** **=0.0174 0.079±0.032** **=0.0143**

Metabolic Factors

Ln glucose — — — — — — — —

Ln glycated hemoglobin — — — — — — — —

Ln HOMA-IR — — — — — — — —

Ln total cholesterol -0.003±0.035 =0.922 — — — — — —

Ln LDL cholesterol — — 0.052±0.034 =0.132 — — — —

Ln HDL cholesterol — — — — -0.051±0.033 =0.121 — —

Ln Triglycerides — — — — — — -0.027±0.038 =0.484

Ln Total/HDL cholesterol — — — — — — — —

Hemodynamic factors

Brachial systolic BP — — — — — — — —

Brachial diastolic BP — — — — — — — —

Brachial pulse pressure — — — — — — — —

Central arterial SBP — — — — — — — —

Central arterial PP — — — — — — — —

Peak aortic flow — — — — — — — —

Stroke volume — — — — — — — —

Stroke volume/BSA — — — — — — — —

Zc — — — — — — — —

TAC — — — — — — — —

Pb — — — — — — — —

Peak P_QxZc_ — — — — — — — —

**Model r^2^** **0.4639** **<0.0001 0.4660** **<0.0001** **0.4662** **<0.0001 0.4643** **<0.0001**

________________________________________________________________________________________________________

The basic model included age, sex, BMI, regular smoking, regular drinking, diabetes mellitus (except for models with glucose, glycated haemoglobin or HOMA-IR due to collinearity), treatment for hypertension, mean arterial pressure (except for models with hemodynamic pressure factors due to collinearity) and heart rate. Subsequent models included those metabolic or hemodynamic factors that were significant in bivariate associations (Table 2). The final model included both metabolic and hemodynamic factors, where the most significant for each were chosen, and total /HDL cholesterol was included as a comparator. Significant associations are shown in bold type. Β, slope; BMI, body mass index; BP, blood pressure; BSA, body surface area; CI, confidence interval; HDL, high-density lipoprotein; HOMA-IR, homeostatic model assessment for insulin resistance; HT, hypertension; LDL, low-density lipoprotein; Ln, natural logarithm; Pb, backward wave pressure; Peak P_QxZc_, component of forward wave pressure generated by the product of peak Q and Zc; PP, pulse pressure; r=Pearson’s correlation coefficient; SBP, systolic blood pressure; Stand, standardised; TAC, total arterial compliance; Zc, aortic characteristic impedance.

**Table S3 continued (2).** Multivariate models showing associations between cardiovascular risk factors and carotid intima-media thickness (IMT) in all participants (n=573).

**IMT versus** Stand. β±sem p-value Stand. β±sem p-value Stand. β±sem p-value Stand. β±sem p-value

________________________________________________________________________________________________________

Model 9 Model 10 Model 11 Model 12

Age **0.581±0.040** **<0.0001** **0.566±0.040** **<0.0001** **0.619±0.038** **<0.0001** **0.552±0.039** **<0.0001**

Sex (male) **0.089±0.038 =0.0189** **0.097±0.037 =0.0086** **0.101±0.038 =0.0075** **0.114±0.036 =0.0019**

BMI 0.071±0.038 =0.064 **0.080±0.037** **=0.0330 0.089±0.038 =0.0201 0.089±0.037 =0.0164**

Regular smoking 0.037±0.035 =0.289 0.039±0.034 =0.259 0.042±0.035 =0.235 0.038±0.034 =0.265

Regular alcohol -0.025±0.034 =0.462 -0.031±0.034 =0.355 -0.029±0.034 =0.399 -0.038±0.033 =0.253

Diabetes mellitus -0.001±0.034 =0.999 0.005±0.034 =0.879 0.003±0.034 =0.941 0.001±0.033 =0.967 Treatment for HT -0.013±0.037 =0.730 -0.015±0.037 =0.677 -0.006±0.038 =0.873 -0.011±0.037 =0.768

Mean arterial pressure **0.102±0.035** **=0.0035**  — — — — — —

Heart rate **0.078±0.032** **=0.0156 0.085±0.032** **=0.0080 0.078±0.032** **=0.0155 0.091±0.032** **=0.0042**

Metabolic Factors

Ln glucose — — — — — — — —

Ln glycated hemoglobin — — — — — — — —

Ln HOMA-IR — — — — — — — —

Ln total cholesterol — — — — — — — —

Ln LDL cholesterol — — — — — — — —

Ln HDL cholesterol — — — — — — — —

Ln Triglycerides — — — — — — — —

Ln Total/HDL cholesterol 0.050±0.035 =0.159 — — — — — —

Hemodynamic factors

Brachial systolic BP — — **0.151±0.036 <0.0001** — — — —

Brachial diastolic BP — — — — 0.052±0.033 =0.118 — —

Brachial pulse pressure — — — — — — **0.177±0.035 <0.0001**

Central arterial SBP — — — — — — — —

Central arterial PP — — — — — — — —

Peak aortic flow — — — — — — — —

Stroke volume — — — — — — — —

Stroke volume/BSA — — — — — — — —

Zc — — — — — — — —

TAC — — — — — — — —

Pb — — — — — — — —

Peak P_QxZc_ — — — — — — — —

**Model r^2^** **0.4658** **<0.0001 0.4723** **<0.0001** **0.4576** **<0.0001 0.4796** **<0.0001**

________________________________________________________________________________________________________

The basic model included age, sex, BMI, regular smoking, regular drinking, diabetes mellitus (except for models with glucose, glycated haemoglobin or HOMA-IR due to collinearity), treatment for hypertension, mean arterial pressure (except for models with hemodynamic pressure factors due to collinearity) and heart rate. Subsequent models included those metabolic or hemodynamic factors that were significant in bivariate associations (Table 2). The final model included both metabolic and hemodynamic factors, where the most significant for each were chosen, and total /HDL cholesterol was included as a comparator. Significant associations are shown in bold type. Β, slope; BMI, body mass index; BP, blood pressure; BSA, body surface area; CI, confidence interval; HDL, high-density lipoprotein; HOMA-IR, homeostatic model assessment for insulin resistance; HT, hypertension; LDL, low-density lipoprotein; Ln, natural logarithm; Pb, backward wave pressure; Peak P_QxZc_, component of forward wave pressure generated by the product of peak Q and Zc; PP, pulse pressure; r=Pearson’s correlation coefficient; SBP, systolic blood pressure; Stand, standardised; TAC, total arterial compliance; Zc, aortic characteristic impedance.

**Table S3 continued (3).** Multivariate models showing associations between cardiovascular risk factors and carotid intima-media thickness (IMT) in all participants (n=573).

**IMT versus** Stand. β±sem p-value Stand. β±sem p-value Stand. β±sem p-value Stand. β±sem p-value

________________________________________________________________________________________________________

Model 13 Model 14 Model 15 Model 16

Age **0.556±0.040** **<0.0001** **0.514±0.042** **<0.0001** **0.629±0.038** **<0.0001** **0.619±0.039** **<0.0001**

Sex (male) **0.102±0.037 =0.0056** **0.127±0.036 =0.0005** **0.114±0.037 =0.0025** **0.111±0.037 =0.0029**

BMI **0.078±0.037 =0.0383 0.089±0.037** **=0.0151 0.101±0.038 =0.0074 0.099±0.038 =0.0089**

Regular smoking 0.036±0.035 =0.299 0.027±0.034 =0.437 0.042±0.035 =0.228 0.044±0.035 =0.207

Regular alcohol -0.031±0.034 =0.358 -0.036±0.033 =0.281 -0.029±0.034 =0.391 -0.032±0.034 =0.246

Diabetes mellitus 0.008±0.034 =0.812 0.008±0.033 =0.812 -0.003±0.034 =0.932 -0.002±0.034 =0.954 Treatment for HT -0.016±0.037 =0.660 -0.018±0.037 =0.615 0.001±0.038 =0.988 0.003±0.038 =0.946

Mean arterial pressure — — — — — — — —

Heart rate **0.095±0.032** **=0.0029 0.122±0.032** **=0.0002 0.081±0.032** **=0.0123 0.083±0.032** **=0.0108**

Metabolic Factors

Ln glucose — — — — — — — —

Ln glycated hemoglobin — — — — — — — —

Ln HOMA-IR — — — — — — — —

Ln total cholesterol — — — — — — — —

Ln LDL cholesterol — — — — — — — —

Ln HDL cholesterol — — — — — — — —

Ln Triglycerides — — — — — — — —

Ln Total/HDL cholesterol — — — — — — — —

Hemodynamic factors

Brachial systolic BP — — — — — — — —

Brachial diastolic BP — — — — — — — —

Brachial pulse pressure — — — — — — — —

Central arterial SBP **0.158±0.037 <0.0001** — — — — — —

Central arterial PP — — **0.215±0.038 <0.0001** — — — —

Peak aortic flow — — — — -0.009±0.032 =0.778 — —

Stroke volume — — — — — — 0.031±0.033 =0.344

Stroke volume/BSA — — — — — — — —

Zc — — — — — — — —

TAC — — — — — — — —

Pb — — — — — — — —

Peak P_QxZc_ — — — — — — — —

**Model r^2^** **0.4724** **<0.0001 0.4848** **<0.0001** **0.4554** **<0.0001 0.4562** **<0.0001**

________________________________________________________________________________________________________

The basic model included age, sex, BMI, regular smoking, regular drinking, diabetes mellitus (except for models with glucose, glycated haemoglobin or HOMA-IR due to collinearity), treatment for hypertension, mean arterial pressure (except for models with hemodynamic pressure factors due to collinearity) and heart rate. Subsequent models included those metabolic or hemodynamic factors that were significant in bivariate associations (Table 2). The final model included both metabolic and hemodynamic factors, where the most significant for each were chosen, and total /HDL cholesterol was included as a comparator. Significant associations are shown in bold type. Β, slope; BMI, body mass index; BP, blood pressure; BSA, body surface area; CI, confidence interval; HDL, high-density lipoprotein; HOMA-IR, homeostatic model assessment for insulin resistance; HT, hypertension; LDL, low-density lipoprotein; Ln, natural logarithm; Pb, backward wave pressure; Peak P_QxZc_, component of forward wave pressure generated by the product of peak Q and Zc; PP, pulse pressure; r=Pearson’s correlation coefficient; SBP, systolic blood pressure; Stand, standardised; TAC, total arterial compliance; Zc, aortic characteristic impedance.

**Table S3 continued (4).** Multivariate models showing associations between cardiovascular risk factors and carotid intima-media thickness (IMT) in all participants (n=573).

**IMT versus** Stand. β±sem p-value Stand. β±sem p-value Stand. β±sem p-value Stand. β±sem p-value

________________________________________________________________________________________________________

Model 17 Model 18 Model 19 Model 20

Age **0.619±0.039** **<0.0001** **0.618±0.037** **<0.0001** **0.621±0.037** **<0.0001** **0.523±0.041** **<0.0001**

Sex (male) **0.114±0.037 =0.0023** **0.125±0.037 =0.0008** **0.119±0.037 =0.0014** **0.134±0.036 =0.0002**

BMI **0.105±0.038 =0.0058 0.103±0.037** **=0.0058 0.102±0.037 =0.0067 0.095±0.036 =0.0092**

Regular smoking 0.044±0.035 =0.213 0.033±0.035 =0.346 0.036±0.035 =0.299 0.031±0.034 =0.361

Regular alcohol -0.032±0.034 =0.353 -0.031±0.034 =0.368 -0.026±0.034 =0.450 -0.034±0.033 =0.307

Diabetes mellitus -0.002±0.034 =0.964 -0.004±0.034 =0.912 -0.003±0.034 =0.932 -0.004±0.033 =0.909 Treatment for HT 0.002±0.038 =0.954 -0.001±0.037 =0.999 -0.008±0.037 =0.838 -0.011±0.036 =0.760

Mean arterial pressure — — — — — — — —

Heart rate **0.082±0.032** **=0.0112 0.086±0.032** **=0.0072 0.088±0.032** **=0.0064 0.125±0.032** **=0.0001**

Metabolic Factors

Ln glucose — — — — — — — —

Ln glycated hemoglobin — — — — — — — —

Ln HOMA-IR — — — — — — — —

Ln total cholesterol — — — — — — — —

Ln LDL cholesterol — — — — — — — —

Ln HDL cholesterol — — — — — — — —

Ln Triglycerides — — — — — — — —

Ln Total/HDL cholesterol — — — — — — — —

Hemodynamic factors

Brachial systolic BP — — — — — — — —

Brachial diastolic BP — — — — — — — —

Brachial pulse pressure — — — — — — — —

Central arterial SBP — — — — — — — —

Central arterial PP — — — — — — — —

Peak aortic flow — — — — — — — —

Stroke volume — — — — — — — —

Stroke volume/BSA 0.028±0.032 =0.381 — — — — — —

Zc — — **0.101±0.031 =0.0012** — — — —

TAC — — — — **-0.069±0.032 =0.0309** — —

Pb — — — — — — **0.213±0.036 <0.0001**

Peak P_QxZc_ — — — — — — — —

**Model r^2^** **0.4560** **<0.0001 0.4653** **<0.0001** **0.4598** **<0.0001 0.4866** **<0.0001**

________________________________________________________________________________________________________

The basic model included age, sex, BMI, regular smoking, regular drinking, diabetes mellitus (except for models with glucose, glycated haemoglobin or HOMA-IR due to collinearity), treatment for hypertension, mean arterial pressure (except for models with hemodynamic pressure factors due to collinearity) and heart rate. Subsequent models included those metabolic or hemodynamic factors that were significant in bivariate associations (Table 2). The final model included both metabolic and hemodynamic factors, where the most significant for each were chosen, and total /HDL cholesterol was included as a comparator. Significant associations are shown in bold type. Β, slope; BMI, body mass index; BP, blood pressure; BSA, body surface area; CI, confidence interval; HDL, high-density lipoprotein; HOMA-IR, homeostatic model assessment for insulin resistance; HT, hypertension; LDL, low-density lipoprotein; Ln, natural logarithm; Pb, backward wave pressure; Peak P_QxZc_, component of forward wave pressure generated by the product of peak Q and Zc; PP, pulse pressure; r=Pearson’s correlation coefficient; SBP, systolic blood pressure; Stand, standardised; TAC, total arterial compliance; Zc, aortic characteristic impedance.

**Table S3 continued (5).** Multivariate models showing associations between cardiovascular risk factors and carotid intima-media thickness (IMT) in all participants (n=573).

**IMT versus** Stand. β±sem p-value Stand. β±sem p-value Stand. β±sem p-value Partial r (95% CI)

________________________________________________________________________________________________________

Model 21 Final Model 1 Final Model 2 Final Model 2

Age **0.569±0.038** **<0.0001** **0.491±0.043** **<0.0001** **0.500±0.042** **<0.0001** **0.448 (0.380 to 0.512)**

Sex (male) **0.109±0.036 =0.0028** **0.115±0.037 =0.0018** **0.122±0.037 =0.0010** **0.138 (0.056 to 0.218)**

BMI **0.090±0.037 =0.0151 0.077±0.037** **=0.0381 0.083±0.037 =0.0248 0.095 (0.012 to 0.176)**

Regular smoking 0.039±0.034 =0.256 0.023±0.034 =0.496 0.028±0.034 =0.413 0.035 (-0.048 to 0.117)

Regular alcohol -0.030±0.033 =0.371 -0.030±0.033 =0.371 -0.028±0.033 =0.404 -0.035 (-0.117 to 0.048)

Diabetes mellitus -0.010±0.033 =0.757 -0.001±0.033 =0.981 -0.013±0.033 =0.700 -0.016 (-0.099 to 0.066) Treatment for HT -0.012±0.037 =0.737 -0.021±0.036 =0.571 -0.013±0.036 =0.715 -0.015 (-0.098 to 0.067)

Mean arterial pressure — — — — — — — —

Heart rate **0.091±0.032** **=0.0043 0.123±0.032** **=0.0001 0.126±0.032** **=0.0001 0.163 (0.081 to 0.242)**

Metabolic Factors

Ln glucose — — — — — — — —

Ln glycated hemoglobin — — — — — — — —

Ln HOMA-IR — — — — — — — —

Ln total cholesterol — — — — — — — —

Ln LDL cholesterol — — — — — — — —

Ln HDL cholesterol — — — — — — — —

Ln Triglycerides — — — — — — — —

Ln Total/HDL cholesterol — — **0.068±0.034** **=0.0488** **0.069±0.034** **=0.0458** **0.084 (0.002 to 0.165)**

Hemodynamic factors

Brachial systolic BP — — — — — — — —

Brachial diastolic BP — — — — — — — —

Brachial pulse pressure — — — — — — — —

Central arterial SBP — — — — — — — —

Central arterial PP — — **0.220±0.038** **<0.0001** — — — —

Peak aortic flow — — — — — — — —

Stroke volume — — — — — — — —

Stroke volume/BSA — — — — — — — —

Zc — — — — — — — —

TAC — — — — — — — —

Pb — — — — **0.218±0.036** **<0.00001** **0.245 (0.166 to 0.321)**

Peak P_QxZc_ **0.166±0.034 <0.0001** — — — — — —

**Model r^2^** **0.4778** **<0.0001 0.4884** **<0.0001** **0.4903** **<0.0001**

________________________________________________________________________________________________________

The basic model included age, sex, BMI, regular smoking, regular drinking, diabetes mellitus (except for models with glucose, glycated haemoglobin or HOMA-IR due to collinearity), treatment for hypertension, mean arterial pressure (except for models with hemodynamic pressure factors due to collinearity) and heart rate. Subsequent models included those metabolic or hemodynamic factors that were significant in bivariate associations (Table 2). The final model included both metabolic and hemodynamic factors, where the most significant for each were chosen, and total /HDL cholesterol was included as a comparator. Significant associations are shown in bold type. Β, slope; BMI, body mass index; BP, blood pressure; BSA, body surface area; CI, confidence interval; HDL, high-density lipoprotein; HOMA-IR, homeostatic model assessment for insulin resistance; HT, hypertension; LDL, low-density lipoprotein; Ln, natural logarithm; Pb, backward wave pressure; Peak P_QxZc_, component of forward wave pressure generated by the product of peak Q and Zc; PP, pulse pressure; r=Pearson’s correlation coefficient; SBP, systolic blood pressure; Stand, standardised; TAC, total arterial compliance; Zc, aortic characteristic impedance.
